# Evolutionary dynamics of sexual size dimorphism in non-volant mammals following their independent colonization of Madagascar

**DOI:** 10.1038/s41598-018-36246-x

**Published:** 2019-02-05

**Authors:** Peter M. Kappeler, Charles L. Nunn, Alexander Q. Vining, Steven M. Goodman

**Affiliations:** 1 Wissenschaftskolleg zu Berlin, Berlin, Germany; 2 Behavioral Ecology and Sociobiology Unit, German Primate Center – Leibniz Institute of Primatology, Göttingen, Germany; 3 Department Sociobiology/Anthropology, University of Göttingen, Göttingen, Germany; 4 Department of Evolutionary Anthropology, Duke University, Durham, NC USA; 5 Duke Global Health Institute, Duke University, Durham, NC USA; 6 Field Museum of Natural History, Chicago, IL USA; 7 Association Vahatra, Antananarivo, Madagascar

## Abstract

As predicted by sexual selection theory, males are larger than females in most polygynous mammals, but recent studies found that ecology and life history traits also affect sexual size dimorphism (SSD) through evolutionary changes in either male size, female size, or both. The primates of Madagascar (Lemuriformes) represent the largest group of mammals without male-biased SSD. The eco-evo-devo hypothesis posited that adaptations to unusual climatic unpredictability on Madagascar have ultimately reduced SSD in lemurs after dispersing to Madagascar, but data have not been available for comparative tests of the corresponding predictions that SSD is also absent in other terrestrial Malagasy mammals and that patterns of SSD changed following the colonization of Madagascar. We used phylogenetic methods and new body mass data to test these predictions among the four endemic radiations of Malagasy primates, carnivorans, tenrecs, and rodents. In support of our prediction, we found that male-biased SSD is generally absent among all Malagasy mammals. Phylogenetic comparative analyses further indicated that after their independent colonization of Madagascar, SSD decreased in primates and tenrecs, but not in the other lineages or when analyzed across all species. We discuss several mechanisms that may have generated these patterns and conclude that neither the eco-evo-devo hypothesis, founder effects, the island rule nor sexual selection theory alone can provide a compelling explanation for the observed patterns of SSD in Malagasy mammals.

## Introduction

Sexual size dimorphism (SSD) provides a striking example of the power of selection to generate differences between males and females of the same species despite shared genetic and developmental histories^1,2^. From this perspective, SSD is best explained as the difference in optimal male and female body size, resulting from independently acting fecundity selection, viability selection and sexual selection^3–5^. For example, increased body size tends to confer a fecundity advantage for females and a competitive advantage for both sexes, whereas it may create or exacerbate viability costs in both sexes, especially during growth and periods of limited resources. Thus, the study of SSD is a central and integrative topic in evolutionary biology because it is closely related to the life history, ecology and behavior of a species.

Explanations for the observed interspecific diversity in SSD are accordingly multifarious. In most endothermic invertebrates and ectothermic vertebrates, fecundity selection on female size appears to be stronger than sexual selection on male size, often resulting in female-biased SSD^6,7^. Conversely, in about 66% of bird^5^ and 45% of mammalian^8^ species, SSD is male-biased because fecundity selection favors small females since their reproductive costs increase with body size^4,5,9–12^ and sexual selection favors larger males because their competitive ability increases with increasing size^5,12–16^. Under certain circumstances, however, sexual selection can also favor smaller males; for example, when speed or agility are more important determinants of male mating success than size and strength^17,18^. Some interspecific variation in mammalian SSD is also explained by differences in body size^5^, phylogenetic signal^19^, or related to dietary divergence between the sexes^20^, suggesting that the causes of interspecific variation in mammalian SSD are more complex than traditionally assumed by singular explanations based on sexual selection theory^16,21^.

Several exceptions to the general mammalian pattern of male-biased SSD have been described^5,22^, including two entire lineages: the Lagomorpha and the primates of Madagascar (Lemuriformes). Lemurs represent an adaptive radiation of more than 120 extant and extinct endemic Malagasy primate species characterized by a lack of male-biased SSD^23,24^, despite wide interspecific variation in body mass (30 g–150 kg), diet, and mating systems^25^. In group-living lemur species, the lack of SSD is accompanied by female dominance, equal adult sex ratios, and female genital masculinization, a combination of traits collectively referred to as the “lemur syndrome”^26,27^.

Just as female-biased SSD in lagomorphs has prompted some investigation^17^, aspects of the lemur syndrome have puzzled evolutionary biologists for decades^28^. Various hypotheses have proposed that the absence of the expected male-biased SSD is either due to some idiosyncrasy of sexual selection^29–31^, masculinized androgen profiles^32,33^, an evolutionary disequilibrium following the Holocene extinction of large lemurs and several top predators^34^, or an adaptive shift in male and female life histories in response to peculiar ecological conditions on Madagascar^35–38^. These ecological idiosyncrasies are ultimately related to the fact that the ecoregions of Madagascar, ranging from arid spiny bush to rainforest, may share unpredictable intra- or inter-annual precipitation compared to other regions of the Old World with similar yearly rainfall profiles^37^. A recent hypothesis combined these earlier notions and attributed the lack of SSD in non-monogamous lemurs to an evolutionary increase in female size, mediated by canalization of developmental consequences of chronic maternal stress in response to low climate predictability (eco-evo-devo hypothesis^27^). Until now, not one of these hypotheses has received unequivocal support, some are difficult to test, and all of them have focused on one of the four living radiations of Malagasy land mammals, namely lemurs.

Here, we extend these findings by (i) broadening the comparative perspective to include the other extant terrestrial mammal lineages of Madagascar and (ii) applying phylogenetic meta- and comparative analysis to investigate evolution on the branches leading to lineages inhabiting Madagascar today. Compared to extralimital taxonomic groups showing behavioral or ecological parallels, several of the endemic Malagasy mammal species have evolved extremely slow or fast life histories^37^, strongly suggesting they have responded to altered ecological conditions with various morphological and physiological adaptations. We therefore ask specifically whether other Malagasy mammals also lack SSD, which would (1) indicate that the absence of male-biased SSD in lemurs is not just an idiosyncrasy of that lineage, and (2) would support the notion that the colonization or ecology of Madagascar have prompted adaptations that are only rarely found in other mammals. We also aim to study in an island setting fine-grained adaptations of body size in both sexes associated with adaptive radiations of ecologically diverse mammals. Because body size influences different traits that are subject to selection, and SSD is the result of evolutionary changes in female size, male size, or both, such analyses can generate hypotheses about sex-specific selective pressures shaping observed patterns.

Body size adaptations following colonization of islands have been studied in other faunas^39–43^, although few have considered islands the size of Madagascar^44^. Only one study has examined sex-specific responses, reporting some evidence for an increase in male-biased SSD (mostly in reptiles and birds) on islands^45^. Because Madagascar experienced numerous climatic vicissitudes in recent geological history, is ecologically very heterogeneous, and all native terrestrial mammals are endemic and have very limited distributions^46,47^, it can be assumed that natural selection had numerous opportunities to influence male and female body size^48^. However, the magnitude and direction of such a shift remain open empirical questions, especially given that the convergence on intermediate body sizes predicted by the island rule were not observed in all orders of terrestrial insular mammals outside of Madagascar^49^.

Amongst Madagascar’s extant fauna, only four orders of non-volant mammals represented in the modern fauna have successfully colonized and radiated into endemic groups dominating the island’s diverse ecosystems^50,51^. Lemurs represent the largest adaptive radiation with about 120 known living and subfossil species dating back to a single successful colonization event about 50 to 60 Myr ago^25^. Carnivorans of the endemic Family Eupleridae are closely allied with mongooses, arrived about 18–24 million years ago, and are represented by 10 extant species^52^. Rodents arrived on Madagascar about 20–25 Myr ago and radiated into 27 currently recognized extant species belonging to the endemic Subfamily Nesomyinae, which is part of broadly distributed African Family Nesomyidae^53^. Finally, tenrecs of the endemic Family Tenrecidae and part of the superordinal clade Afrotheria, a largely Afro-Malagasy radiation, have been present on Madagascar since about 30–56 Myr ago and are represented by 32 known extant species^53^. Amongst the other recent mammals of Madagascar, bats have colonized Madagascar multiple times^51^ but are not considered here because their different life history traits may affect body size and SSD. Further, we do not include recently extinct lemurs, pygmy hippos and the enigmatic Order Bibymalagasia in our analyses, nor do we examine human-introduced *Rattus*, *Mus*, *Suncus* and *Potamochoerus*.

Evidence for the existence of a combination of traits characterizing the lemur syndrome in the other Malagasy mammalian lineages is virtually absent because less than a handful of them have been subjected to behavioral field studies of known individuals. As a result, the mating systems of Malagasy non-primate mammals remain largely unknown, preventing a formal test of the effects of sexual selection on SSD. However, pair-living has only been reported for the giant jumping rat *Hypogeomys antimena*^54^, indicating that monogamy among non-primate Malagasy mammals is rare. In euplerid carnivorans, *Cryproprocta ferox*^55^ and *Mungotictis decemlineata*^56^, the mating system is clearly promiscuous, and in *Galidictis grandidieri*, there is indirect evidence of the absence of pair-living^57^. To the extent that this is known (Goodman, unpublished data), tenrecs, rodents and other carnivorans breed once a year during particular seasons; these conditions are generally conducive to promiscuity. Among lemurs, pair-living is much more common^58^, but (male-biased) SSD is also absent in species with other mating systems^27^.

With new body mass data collected on Malagasy mammals from thousands of individuals handled in the wild, combined with data from non-Malagasy mammals, we use three phylogenetic methods to investigate evolutionary changes in body size and SSD associated with the independent colonization of Madagascar by these four independent lineages. First, we use phylogenetic meta-analysis to investigate whether Malagasy species generally lack dimorphism. Second, we use phylogenetic generalized least squares approaches to investigate the effect of living on Madagascar for SSD in the individual lineages, and across all mammals for which data on SSD are available. Finally, we used a model which incorporates drift, stabilizing selection, and shifts in adaptive optima (the Ornstein-Uhlenbeck model^59^). Because any changes in SSD following the colonization of Madagascar could be due to either an increase in female size or a decrease in male size, and because the post-colonization changes in body size might vary as a function of both taxonomic affiliation and absolute body size^49^, we explore evolutionary changes in male and female body size within each of our focal lineages. We do not test the effects of phenological and climatic variability on SSD directly because of a lack of relevant data (but see^38^). Instead, we describe patterns of SSD in Malagasy mammals and investigate evolutionary dynamics in SSD and body mass following the colonization of Madagascar.

## Results

### Sexual size dimorphism within Malagasy mammals

**Figure 1:**
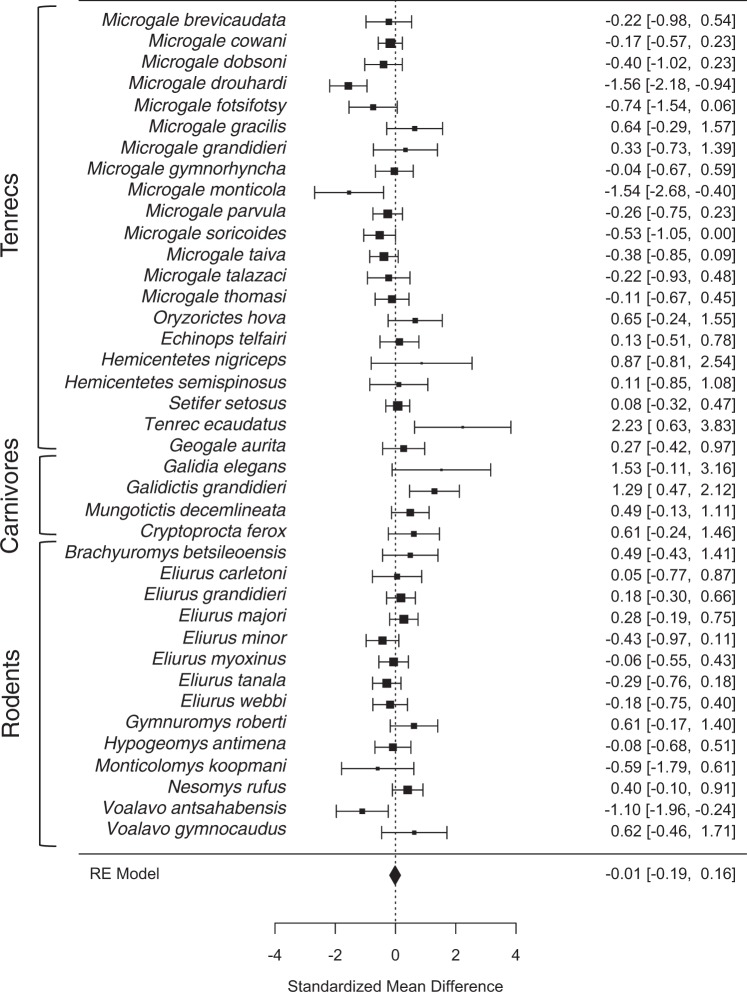
Patterns of SSD in Malagasy non-primate mammals. Depicted are results of a meta-analysis treating each species as a separate study, with the goal to infer the overall effect size of “sex” on body mass. Effect sizes are represented as standardized mean difference, with positive values indicating male-biased sexual dimorphism, and their 95% confidence intervals. The overall effect size (and confidence intervals) is shown at the bottom as a diamond. For a similar analyses limited to non-primate mammals for which ohylogenetic information is available, see Supplementary Fig. S5.

In a meta-analysis that used all available data (n = 39 species), the overall effect size was small (−0.012), with 95% confidence intervals that included zero (−0.19 to 0.16), suggesting an absence of sexual dimorphism in the Malagasy mammals for which we have new intraspecific data to analyze. These non-phylogenetic results are depicted graphically as a forest plot in Fig. 1.

Just as phenotypic characteristics can covary with phylogeny and necessitate a phylogenetic comparative analysis, effect sizes can, too, calling for caution in this non-phylogenetic meta-analysis. Indeed, in an analysis of phylogenetic signal in effect sizes across species that could be placed on phylogeny, we found that the maximum likelihood estimate of λ (Pagel’s phylogenetic signal) was 0.74, which was statistically different from zero (p = 0.024). On this basis, we conducted a phylogenetic meta-analysis with the branch lengths from Fritz *et al*.^60^ and after transforming those branches by the estimated λ. The phylogenetic meta-analysis produced an Akaike Information Criterion (corrected for small sample size; AICc) of 50.38 (n = 29 species), which was similar to that produced with the λ-transformed tree (AICc = 50.69), but much smaller than without phylogeny (AICc = 68.65, analyses not shown), and thus strongly supportive of using phylogenetic methods. Results were similar in the two phylogenetic models, with 95% confidence intervals that included zero (without λ-transformation: mean effect = 0.31, CI = −0.26 to 0.88, see Supplementary Fig. S5; with λ-transformation: mean effect = 0.26, CI = −0.17 to 0.70). Thus, phylogenetic analysis appeared to increase the effect size, but also widened the confidence intervals considerably.

Of the species of Malagasy non-primate mammals for which we present new data, females are heavier than males in three species (Fig. 1). In contrast, only one species of Tenrecidae (*Tenrec ecaudatus*) exhibits male-biased SSD; this pattern has been previously noted based on morphological data^53^. However, given that this species undergoes massive seasonal fluctuations in body mass due to extended annual hibernation, the SSD value is difficult to interpret, and we did not include it in our comparative analyses because of uncontrolled seasonal variation in our sample. Among the previously studied species, only *Galidictis grandidieri* exhibits significant male-biased SSD^57^. Thus, as in lemurs, the vast majority of other Malagasy mammals is characterized by no or slightly female-biased SSD.

### Evolution of Sexual Size Dimorphism on Madagascar: BayesModelS-PGLS Analyses

**Figure 2:**
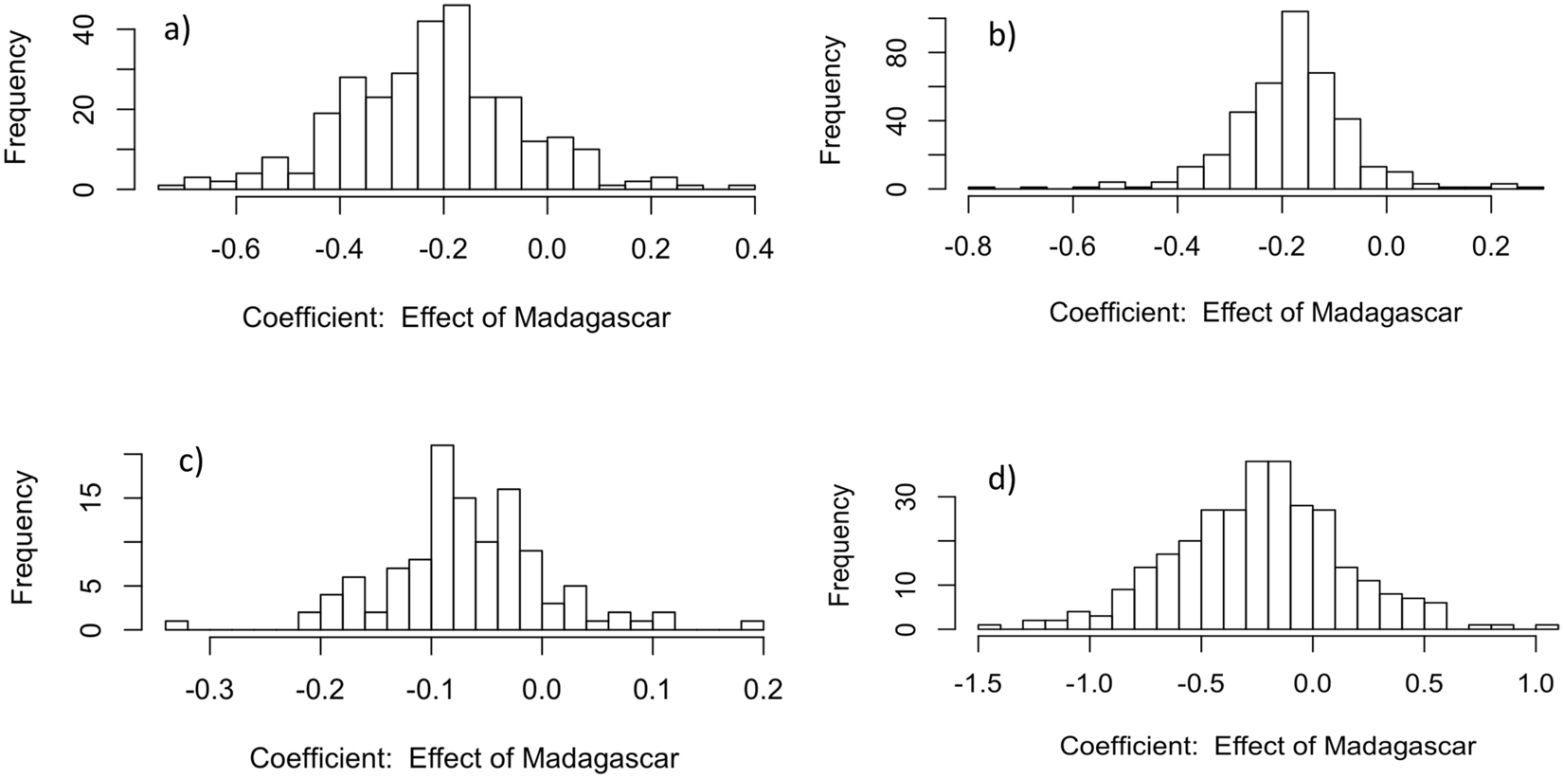
Effect of “location” (Madagascar or non-Madagascar) on SSD in four mammalian lineages. Depicted are posterior probability distributions of the regression coefficients of phylogenetic generalized least squares models, including Bayesian model selection, to assess what proportion of models the regression coefficient “location” was included in the model. A negative coefficient indicates less SSD along the Malagasy lineage. (**a**) afrotherians (tenrecs), (**b**) primates (lemurs), (**c**) rodents, and (**d**) carnivorans.

In phylogeny-based analyses that account for shared ancestry of tenrecs and their close relatives (Afrotheria), we found some evidence for reduced SSD in tenrecs: the effect of Madagascar on SSD was negative when examined in a simple model (*β*_*location*_ = −0.22, Fig. 2a). This coefficient was included in 29.8% of the 1000 Markov-Chain-Monte Carlo (MCMC) samples, and when included, was typically negative (89.6% of the samples), but this effect disappeared when including female body mass in the model (*β*_*location*_ = −0.073, included in 15.4% of the 1000 MCMC samples, and when included, was negative in 63% of the samples). In lemurs, phylogenetically controlled analyses were also indicative of an effect of *β*_*location*_. Thus, *β*_*location*_ was estimated as −0.18 (Fig. 2b); it was included in 39.7% of MCMC samples and less than zero in 95.2% of those samples. Controlling for female body mass again produced similar results (*β*_*location*_ = −0.18, included in 43.4% of the 1000 MCMC samples, and when included, was negative in 95.9% of the samples). In endemic Malagasy rodents, support for the effect of location was weaker; *β*_*location*_ was estimated as −0.066, but was included in only 11.6%. This coefficient was often negative in those posterior samples (87.1%, Fig. 2c). Results were qualitatively similar when controlling for female body mass. In phylogenetic analyses of carnivorans, weak indications of a slight decrease in SSD were again found (Fig. 2d; *β*_*location*_ = −0.24, with *β*_*location*_ included in 30.6% of the MCMC samples, and 75.2% of the estimates less than zero). Controlling for female body mass produced qualitatively similar results.

Finally, we investigated the effects of body mass and residence in Madagascar on SSD across all mammals for which we had SSD data and corresponding phylogenetic information (n = 1379 species). In phylogenetic analyses of the full-mammal dataset using Phylogenetic Generalized Least Squares (PGLS), the results were not conclusive, with three very different models that were within two AIC units of the best model. The best supported model included only *β*_*female_mass*_ and estimated λ (AIC = 914.2, *β*_*female_mass*_ = 0.068, t_1377_ = 3.48, p = 0.0005). The second-best model included *β*_*female_mass*_ and *β*_*location*_ and estimated λ (AIC = 915.4, *β*_*female_mass*_ = 0.067, t_1376_ = 3.44, p = 0.0006; *β*_*location*_ = −0.11, t_1376_ = −0.92, p = 0.36). The third best model represented a null model (no coefficients) that estimated κ (AIC = 915.8). In the two best models that estimated λ, confidence intervals on λ excluded 0 (best supported model: CI = 0.71 to 0.84; second best model: 0.71 to 0.84), and in the third best model that estimated κ, confidence intervals on κ excluded 1 (CI = 0 to 0.111). Thus, the analyses clearly demonstrate evidence for phylogenetic signal, but failed to convincingly support the prediction of the eco-evo-devo hypothesis.

### Evolution of Sexual Size Dimorphism on Madagascar: Bayou-OU Analyses

**Figure 3:**
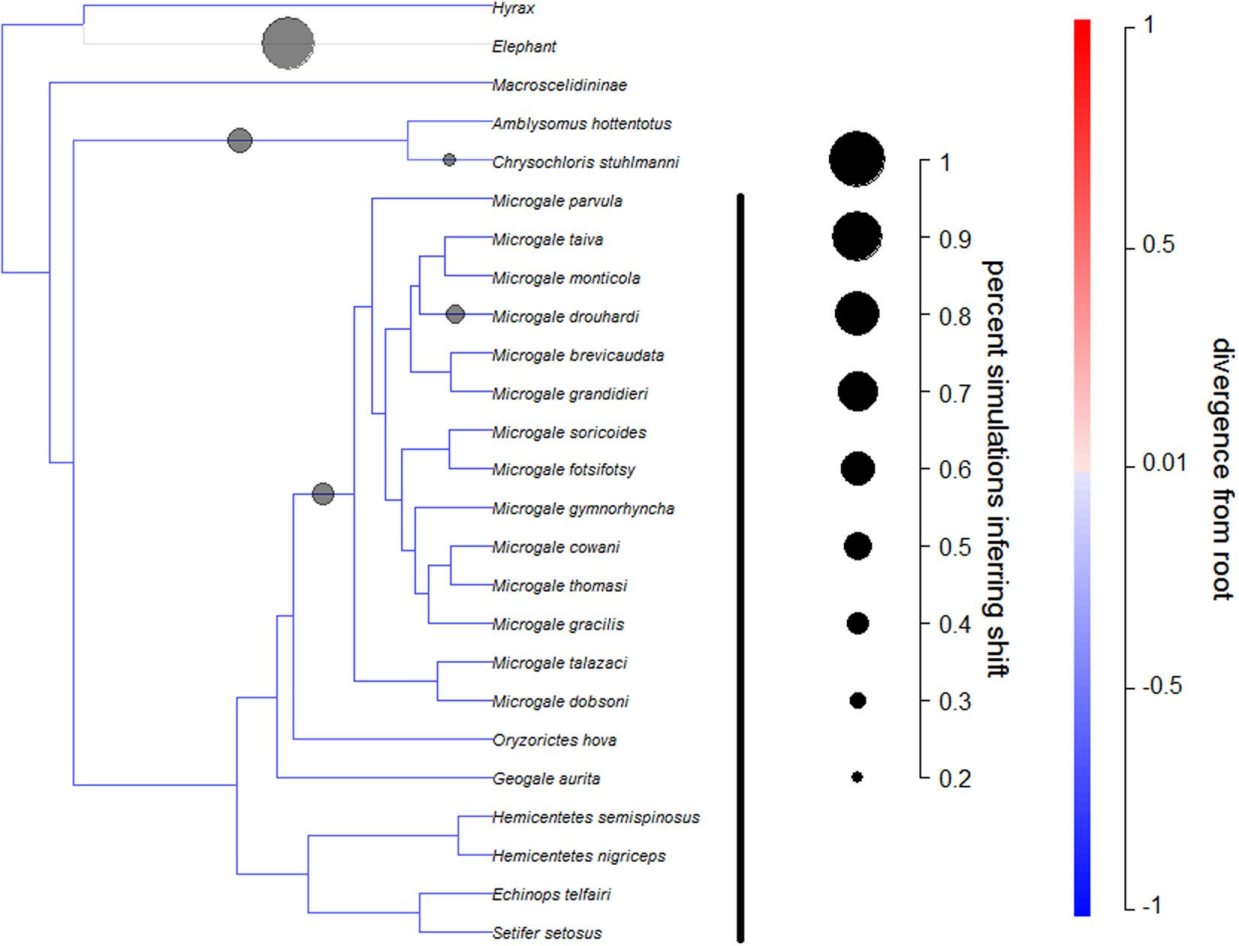
Modeling adaptive regimes of SSD in afrotherians. Using bayou analysis to characterize adaptive regimes across a phylogenetic tree, we assessed whether the regime has undergone a negative shift along the most basal branches leading to the Malagasy lineage and/or a positive shift in its sister clade on mainland Africa. Darker branches represent larger SSD (males are bigger relative to females), while the size of the circles indicate support for changes to occur along the lineage (circles are only included for branches with changes in more than 20% of the models). The bayou analyses revealed that changes in SSD on the root branch leading to Malagasy lineages were negative, and support for this change was strong for afrotherians.

Using the Ornstein-Uhlenbeck (OU) model implemented in bayou, we found some indications of selective regime shifts on the branch of the phylogeny leading to the Malagasy genus *Microgale*, but not on the lineage to the tenrecs themselves (Fig. 3). The average change in SSD along this branch was negative (mean change = −0.117, sd = 0.171), with about 13% of models in the posterior sample including a shift on this branch. There was stronger support for an increase in SSD in mainland golden moles (mean change = 0.172, sd = 0.120), with 46% of models inferring a change along this branch. Like other phylogenetic methods, bayou may have difficulty distinguishing between an increase on one branch and a decrease on a sister branch; thus, these increases may reflect decreases along the branch leading to the Malagasy lineages. The basal branches for mainland golden moles and *Microgale* receive the 2^nd^ and 3^rd^ most support on the tree, behind elephants.

**Figure 4:**
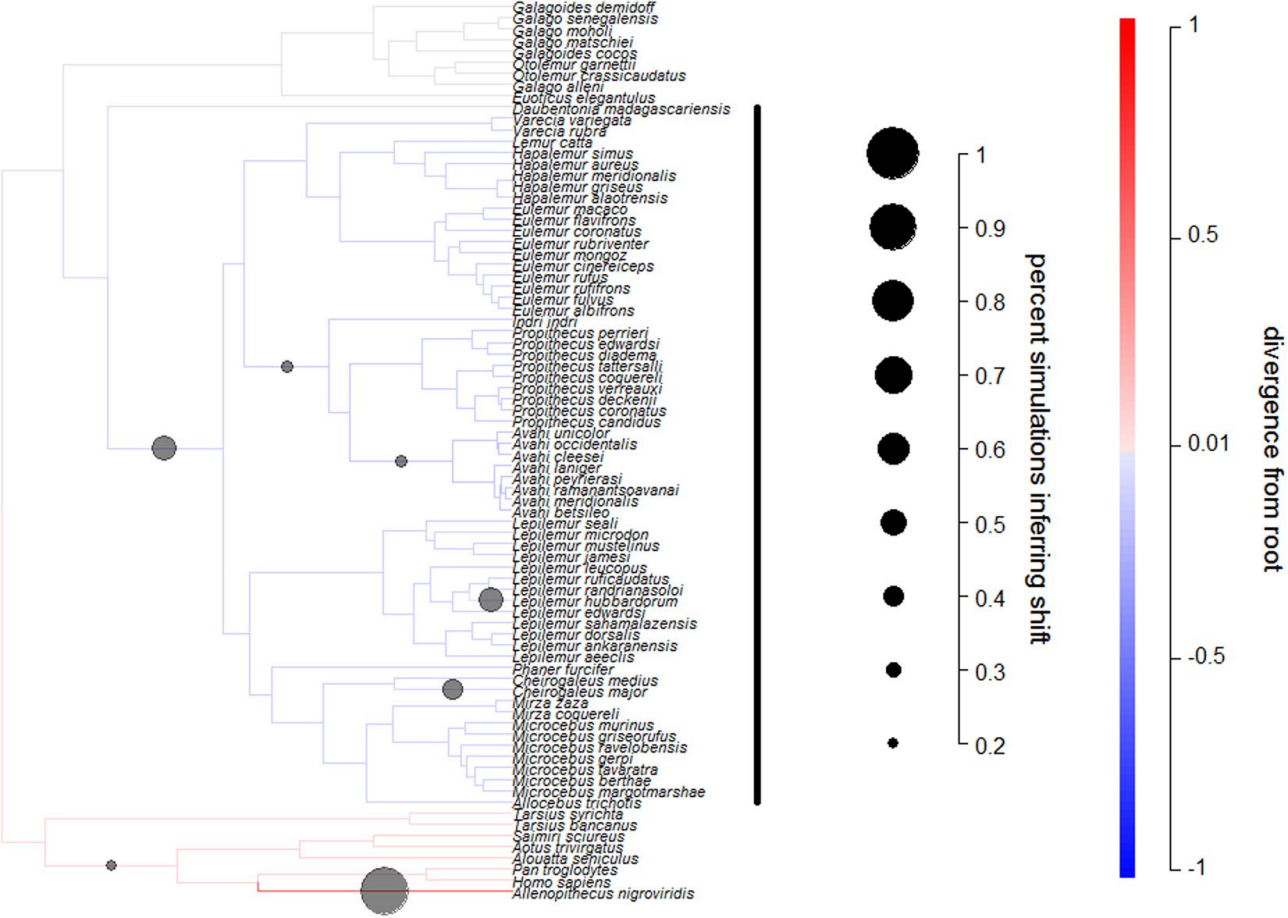
Modeling adaptive regimes of SSD for primates. Darker branches represent larger SSD (males are bigger relative to females), while the size of the circles indicate support for changes to occur along the lineage (circles are only included for branches with changes in more than 20% of the models). The bayou analyses revealed that changes in SSD on the root branch leading to Malagasy lineages were negative, and support for this change was strong for primates.

The bayou analysis for primates showed some indications of a decrease in SSD on the branch leading to lemurs (Fig. 4), with 16% of models in the posterior containing a shift along this branch (mean = −0.146, sd = 0.051). Additionally, there was stronger support for a decrease in SSD on the branch leading to all lemurs but *Daubentonia madagascariensis*, with 48% of models on this branch containing a shift (mean shift = −0.142, sd = 0.044). Across the tree, these branches received the 2^nd^ and 9^th^ most support for a shift.

**Figure 5:**
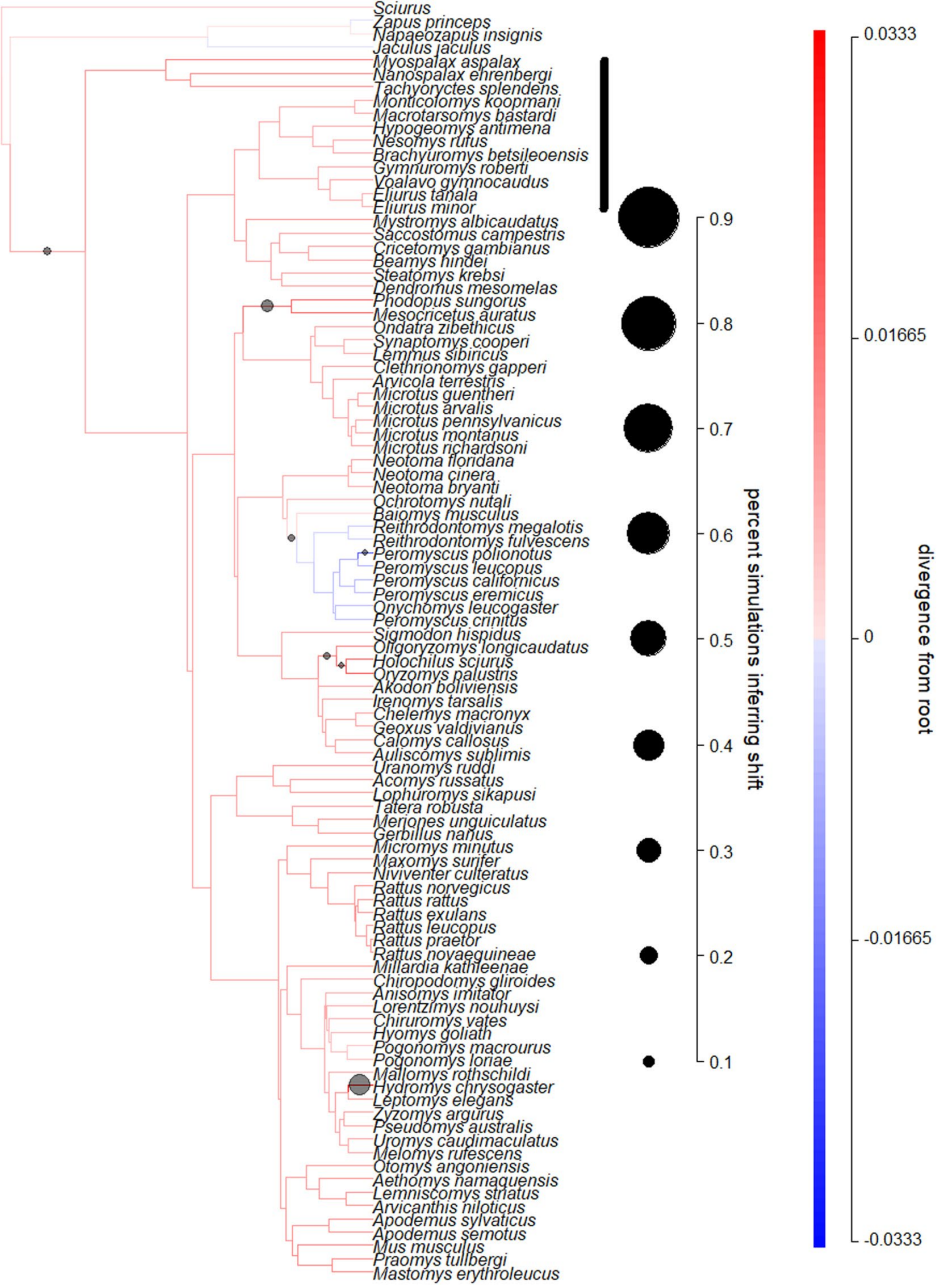
Modeling adaptive regimes of SSD in rodents. Darker branches represent larger SSD (males are bigger relative to females), while the size of the circles indicate support for changes to occur along the lineage (circles are only included for branches with changes in more than 20% of the models). The bayou analyses revealed that changes in SSD on the root branch leading to Malagasy lineages were negative, but support for this change was not strong for rodents.

**Figure 6:**
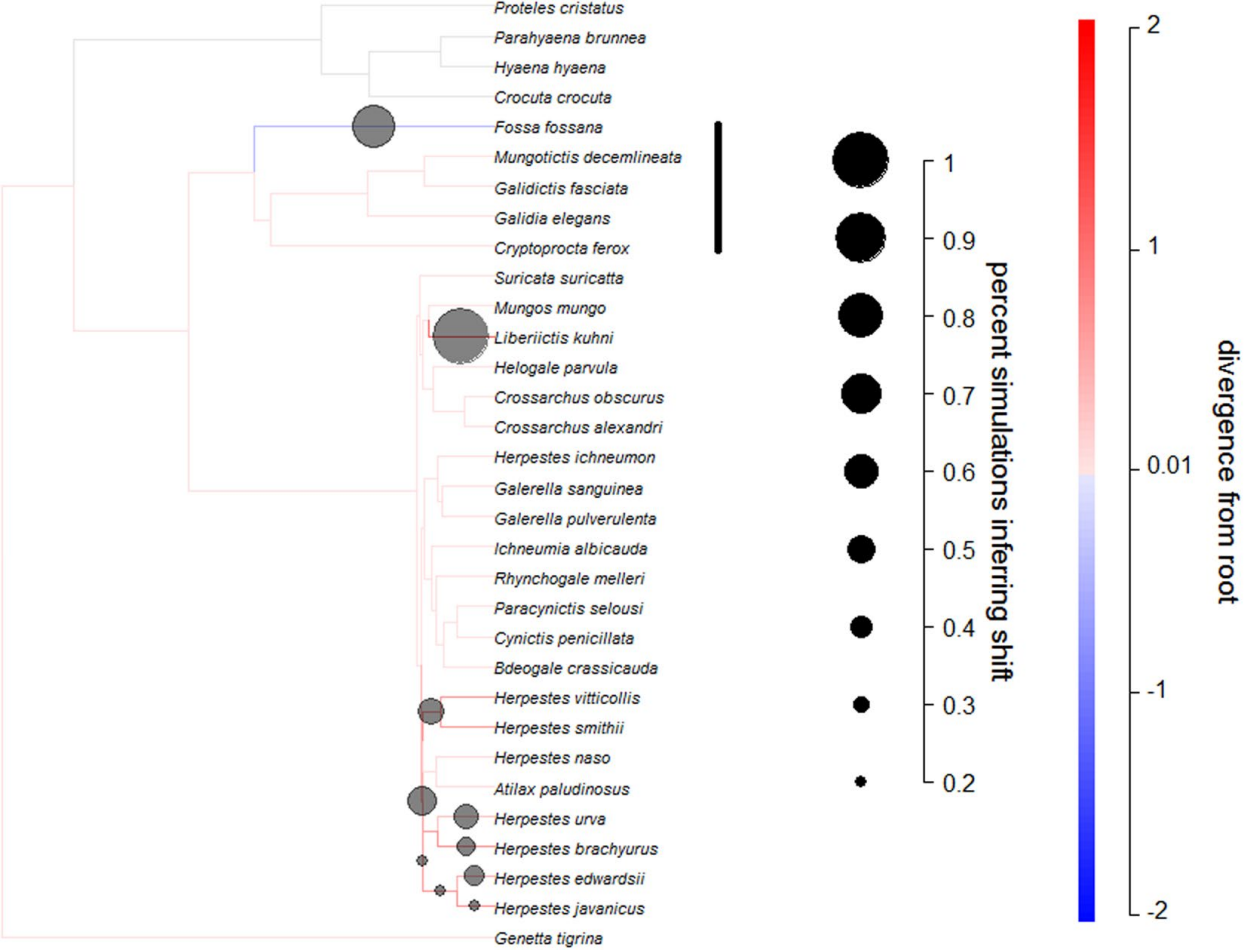
Modeling adaptive regimes of SSD in carnivorans. Darker branches represent larger SSD (males are bigger relative to females), while the size of the circles indicate support for changes to occur along the lineage (circles are only included for branches with changes in more than 20% of the models). The bayou analyses revealed that changes in SSD on the root branch leading to Malagasy lineages were negative, but support for this change was not strong for carnivorans.

Bayou revealed little evidence of regime shifts along basal branches for rodents (Fig. 5). Branches leading to the Malagasy lineage contained a shift in only 2% of models, and these tended to be negative (mean = −0.057, sd = 0.058). In carnivorans, we found evidence for a reduction in SSD in the *Fossa fossana* lineage, but not in other Malagasy carnivorans (Fig. 6). The branch leading to the Malagasy carnivorans only had a shift in 4% of models, while the *F. fossana* branch had shifts in 76% of models (mean shift = −0.514, sd = 0.142).

Thus, across all four analyses, changes in SSD on the branch leading to Malagasy lineages were consistently negative. Evidence for this change was relatively stronger in the primates than for tenrecs, with no evidence in the rodent and carnivoran analyses.

### Sex-specific body mass evolution

For body mass, we found less support for consistent changes in either female or male body mass across the four lineages. BayesModelS revealed support for a decline in female body mass for tenrecs (*β*_*location*_ was included in 82.6% of MCMC samples, and among those included, 96.9% of the coefficients were negative, Supplementary Fig. S6a). A similar pattern was found for males (*β*_*location*_ was included in 83.8% of MCMC samples, and among those included, 97% of the sample of non-zero coefficients were negative, Supplementary Fig. S6b). Bayou analyses of adaptive regimes in female and male body mass revealed more striking shifts within subclades of both Malagasy and non-Malagasy species, as compared to lineages leading to the Malagasy species (Supplementary Figs S6c,d).

In BayesModelS analyses of primate female mass, we found that *β*_*location*_ was on average modestly positive (Supplementary Fig. S7). In 41% of the models from the MCMC analysis, *β*_*location*_ was included, and it was estimated to be positive in 78.4% of those samples. For males, in 34% of the models from the MCMC analysis, *β*_*location*_ was included, and it was estimated to be positive in 78.7% of those samples. Unfortunately, bayou failed to effectively model these data, with all analyses getting stuck in various regions of low maximum-likelihood and failing to reach convergence. Though these problems can sometimes be solved by developing more informed priors or altering the MCMC parameters (Ho & Ané 2014), we were unable to do so in this instance.

We found no support for changes in body mass along the lineage leading to Madagascar in rodents. BayesModelS analyses revealed no support for increased female body mass in Malagasy rodents (Supplementary Fig. S8a; *β*_*location*_ = −0.32, with *β*_*location*_ included in 30% of the 1000 MCMC samples, with 79.7% of these coefficients negative). Similar results were found for males in PGLS analyses (*β*_*location*_ = −0.35, with *β*_*location*_ included in 32% of the 1000 MCMC samples, with 82% of these coefficients estimated to be negative) and in bayou (Supplementary Fig. S8b,c). We also found no compelling effects of location on female body mass in BayesModelS (Supplementary Fig. S9a) or effects for either sex in bayou analyses for carnivorans (Supplementary Fig. S9b,c).

## Discussion

This study was motivated by observations of very low SSD in Malagasy primates. Phylogenetically-controlled comparative analyses suggest that after their independent colonization of Madagascar, SSD decreased in primates and tenrecs, but not in the other lineages or when analyzed across all mammals. We found no evidence for consistent sex-specific changes in adult body mass producing these patterns of SSD among Malagasy mammals. The widespread lack of SSD and its taxon-specific dynamics may have different causes. We discuss the most likely evolutionary, ecological and phylogenetic factors contributing to this pattern in what follows.

The observed pattern of low SSD among Malagasy mammals could be due to founder effects, if the respective last common ancestor of the four lineages lacked SSD and the descendent species did not subsequently deviate from this pattern. However, the living members of the closest African sister lineages of the lemurs, rodents and tenrecs, exhibit on average male-biased SSD, making it highly unlikely that the founding species colonizing Madagascar were characterized by a lack of SSD. The situation is different in carnivorans, where the available data indicate that the euplerids tend to exhibit low average levels of SSD that resemble patterns observed among African Herpestinae more than those among the Mungotinae. Because both the number of species and the samples sizes within species for carnivorans are generally low, this pattern should be regarded as preliminary. Nonetheless, it is possible that euplerids maintained the average degree of SSD exhibited by their last common ancestor that colonized Madagascar, and, importantly, SSD did not change as predicted by the island rule. Thus, the average level of SSD exhibited significant reductions in two out of four mammalian lineages after colonizing Madagascar, suggesting that SSD is evolutionarily plastic, but that lineage-specific effects modulate this plasticity.

Several previous studies revealed an increase in body mass following the colonization of islands from nearby continental areas^49^, but several other studies, including those investigating carnivorans, failed to do so^45,61^. In principle, the dynamics of body size evolution upon colonizing new habitats may liberate both male and female body size to change independently, thereby providing a mechanism for adjusting levels of SSD. A common pattern for small mammals is to undergo increases in both average size and SSD upon colonizing islands^45,62^, the latter of which must be due to a disproportionate increase in male size. However, as in previous studies of other island carnivorans^61^, we found no consistent effects of changes in either male or female body mass of the relatively small Malagasy carnivorans upon colonizing Madagascar.

In afrotherians and primates, we found some evidence that the average SSD of the Malagasy lineages was reduced; in lemurs, this pattern was “weak”. Importantly, however, the observed changes in SSD are in the opposite direction from those predicted by the island effect, and there was little evidence for significant changes in either male or female size, except for lemurs. Thus, the observed evolutionary changes in SSD associated with the colonization of Madagascar cannot be fully explained by the expected island effect.

Among mammals, males have higher potential reproductive rates than females because of the physiological constraints of internal gestation and obligatory female lactation. Male fitness is therefore primarily limited by access to fertile females ready to mate, subjecting males to strong intrasexual selection. SSD in most non-monogamous mammals is biased in favor of males^5,8,14,16^. This pattern arises because large body size, sometimes in combination with species-specific weapons such as antlers or elongated teeth, confers an advantage in male-male contests that determine male lifetime reproductive success in the context of mating and infanticide protection^63^.

The mating systems of Malagasy non-primate mammals remain largely unknown, preventing a formal test of the effects of the mating system on SSD. Because at least mild male-biased SSD is common among the African sister lineages of lemurs, tenrecs, and rodents, similar patterns ought to be expected among their Malagasy relatives with similar sizes, ecologies and presumably mating systems if sexual selection was a main driver of SSD, however. Based on the results of the present analyses, we conclude that the expected effects of sexual selection on SSD are not discernible in Malagasy primates, rodents, and tenrecs, however. In the next section, we address possible reasons for the deviation of Malagasy carnivorans.

Preliminary data indicate that Madagascar is characterized by more pronounced climatic unpredictability than adjacent mainland Africa^37^, which translates into resource unpredictability. Specifically, pronounced seasonality, coupled with strong inter-annual climatic variation, is supposed to create conditions that result in resource constraints for reproductive females, favoring adaptations that either maximize energy intake or minimize energy expenditure. The eco-evo-devo hypothesis assumes that, if females of primary consumer species are stressed during reproduction year after year, an evolutionary increase in female body size is expected, gradually reducing the degree of SSD^27^.

There was no statistical support for the predicted evolutionary increase in female mass in female lemurs, rodents and carnivorans, and there was even a tendency for a reduction of female body mass for tenrecs. Carnivorans may be ecologically buffered from short-term variation and unpredictability of their main food resources because they feed on multiple prey species, thus removing them by one trophic level from the direct effects of unpredictability in resource availability. Several tenrecid species, which also have a carnivorous diet, respond to periods of food scarcity and unfavorable seasons with torpor and hibernation^64^, which may buffer them from the effects of pronounced intra-annual variation in food availability. For the herbivorous and frugivorous rodents, there is no obvious explanation why they may suffer less from resource competition, thus failing to support this assumption of the eco-evo-devo hypothesis.

The weak signal for an evolutionary increase in lemur male mass was not predicted by the eco-evo-devo hypothesis. As this change is also unrelated to variation in the intensity of sexual selection, it might be due to the fact that lemurs were the first of the extant mammal groups to colonize Madagascar, where a rapid adaptive radiation into many available niches occurred. It is therefore not surprising that we detected weak signals of evolutionary increases in body mass in lemurs of both sexes.

In conclusion, this study revealed that a lack of SSD characterizes most species of Malagasy land mammals, and provides suggestive evidence that SSD decreased in lemurs and tenrecs. The lemur syndrome may therefore also apply to tenrecs. Endemic tenrecids and nesomyine rodents exhibit similar patterns, and all three clades are derived from African lineages exhibiting mild male-biased SSD. Because the vast majority of the Malagasy species of these three groups presumably also have non-monogamous mating systems, the expected effects of sexual selection on SSD must have been checked by other selective factors. Patterns of SSD and body size evolution do not consistently follow predictions of the island rule, and the eco-evo-devo hypothesis may explain some of the patterns of SSD and body size evolution in lemurs and tenrecs. Lineage-specific adaptive responses to resource unpredictability invoke ecological factors as powerful determinants of SSD that may have been overlooked in many previous studies of more strongly dimorphic species, but additional data on climatic and phenological variability in southern Africa are required for a conclusive test of this assumption of the eco-evo-devo hypothesis. Thus, a massive advancement in the quantity and quality of available morphometric data for a wide variety of Malagasy mammals, as well as new sophisticated comparative methods, currently fail to fully explain the evolutionary dynamics of SSD, which are clearly more complex than previously thought.

## Methods

### Body mass data

We used a combination of unpublished and published data on body mass to quantify the degree of sexual dimorphism in 102 species of Malagasy mammals and 67 species of extralimital taxa, that may be sister groups to their Malagasy counterparts. Original data come from 931 adult males and 701 females in the Malagasy Tenrecidae, Eupleridae and Nesomyinae, based on captures during field expeditions by SMG (Table 1). All methods were carried out in accordance with relevant guidelines and regulations and with a corresponding research permit issued by the Ministry of the Environment, Water and Forests of Madagascar. Only information from adult, non-gestating and non-lactating individuals were used herein. Data on *Cryptoprocta ferox*, *Mungotictis decemlineata*, *Galidictis grandidieri* and *Hypogeomys antimena* were extracted from published sources (preferring studies with a larger sample size) or from Lindenfors *et al*.^5^. Previously published data for lemurs were updated from the literature (Supplementary Table S1). Data on non-Malagasy mammals (Supplementary Tables S2 and S3) were also obtained from published sources^5^, and we obtained unpublished data from colleagues with field projects in Africa.

**Table 1:** Body mass (g) and sexual size dimorphism (SSD) in terrestrial Malagasy mammals.

Species	F	SD	N	M	SD	N	SSD	p	Reference
**Tenrecidae: Oryzorictinae**									
*Microgale brevicaudata*	9.7	1.16	8	9.4	1.52	42	0.96	ns	this study
*Microgale cowani*	12.7	1.83	58	12.4	1.71	41	0.97	ns	this study
*Microgale dobsoni*	28.9	5.39	18	26.9	4.38	23	0.93	ns	this study
*Microgale drouhardi*	12.5	1.79	23	10.1	1.26	30	0.76	<0.001	this study
*Microgale fotsifotsy*	9.2	1.90	8	7.8	1.75	29	0.83	ns	this study
*Microgale gracilis*	22.2	2.00	7	24.0	2.74	14	1.08	ns	this study
*Microgale grandidieri*	8.3	1.70	5	9.0	2.08	11	1.08	ns	this study
*Microgale gymnorhyncha*	17.7	2.87	23	17.6	2.61	17	0.99	ns	this study
*Microgale monticola*	14.0	0.63	6	12.8	0.79	10	0.91	0.007	this study
*Microgale parvula*	3.2	0.47	27	3.1	0.45	40	0.96	ns	this study
*Microgale soricoides*	19.3	2.42	29	18.0	2.57	28	0.93	0.049	this study
*Microgale taiva*	12.3	2.00	32	11.7	1.25	39	0.95	ns	this study
*Microgale talazaci*	39.7	4.23	16	38.9	2.51	15	0.98	ns	this study
*Microgale thomasi*	22.1	3.51	19	21.7	2.46	34	0.98	ns	this study
*Oryzorictes hova*	33.0	4.88	6	37.2	6.56	27	1.13	ns	this study
**Tenrecidae: Tenrecinae**									
*Echinops telfairi*	99.9	17.82	15	102.4	19.29	24	1.03	ns	this study
*Hemicentetes nigriceps*	98.0	6.56	3	111.0	15.59	3	1.13	ns	this study
*Hemicentetes semispinosus*	107.6	16.01	5	109.5	16.72	24	1.02	ns	this study
*Setifer setosus*	218.8	52.17	46	223.0	57.62	52	1.02	ns	this study
Tenrec ecaudatus*	658.3	104.10	6	1027.5	203.37	4	1.56	0.005	this study
**Tenrecidae: Geogalinae**									
*Geogale aurita*	7.0	1.34	15	7.3	1.02	17	1.05	ns	this study
**Eupleridae**									
*Galidia elegans*	642.5	102.53	2	820.4	108.16	10	1.28	ns	this study
*Galidictis grandidieri*	1400.0	118.4	10	1650.0	213.2	20	1.18	0.01	58
*Mungotictis decemlineata*	538.7	52.04	19	560.7	35.37	22	1.04	ns	57
*Cryptoprocta ferox*	7243.8	855	8	8233.3	1783	18	1.14	ns	56
**Nesomyidae: Nesomyinae**									
*Brachyuromys betsileoensis*	104.9	13.81	7	113.8	18.90	14	1.09	ns	this study
*Eliurus carletoni*	80.3	13.55	12	81.0	13.64	11	1.01	ns	this study
*Eliurus grandidieri*	51.8	4.19	33	52.6	4.81	35	1.02	ns	this study
*Eliurus majori*	98.9	14.44	27	103.9	19.29	50	1.05	ns	this study
*Eliurus minor*	38.1	4.46	24	36.2	4.07	30	0.95	ns	this study
*Eliurus myoxinus*	65.6	7.62	36	65.1	5.59	29	0.99	ns	this study
*Eliurus tanala*	91.1	15.73	33	87.0	12.41	37	0.95	ns	this study
*Eliurus webbi*	80.0	9.14	25	78.4	9.69	22	0.98	ns	this study
*Gymnuromys roberti*	126.1	13.80	14	136.8	20.06	12	1.09	ns	this study
*Hypogeomys antimena*	1120.0	120	25	1110.0	110	19	0.99	ns	55
*Macrotarsomys ingens*	69		1	72		1	1.04	—	this study
*Monticolomys koopmani*	26.4	1.11	4	24.7	2.99	9	0.93	ns	this study
*Nesomys rufus*	156.6	16.66	28	163.3	15.89	33	1.04	ns	this study
*Voalavo antsahabensis*	21.8	2.60	11	19.5	1.48	13	0.88	0.01	this study
*Voalavo gymnocaudus*	21.3	2.44	6	22.8	2.10	8	1.07	ns	this study

Mean adult body mass of adult females (F) and males (M) along with the respective standard deviation(SD) and sample size (N). Sexual size dimorphism (SSD) is expressed as the two-step ratio. P-values correspond to two-tailed t-tests testing for significant sex differences in mean body mass. *Excluded from comparative analyses because of uncontrolled seasonal variation.

We selected species for inclusion based on phylogenies that were available for the four Malagasy groups and their close relatives, which we here call “comparison groups” and included several outgroups to better assess evolutionary dynamics of SSD evolution following the colonization of Madagascar. We preferred phylogenetic trees that were inferred using Bayesian methods, and for which we could obtain a posterior distribution of trees for incorporating phylogenetic uncertainty^65^. For carnivorans, we obtained a tree block of 100 dated phylogenies from 10kTrees (Version 3^66^), including all species of the families Eupleridae, Herpestidae, Hyaenidae, and 1 species from the Viverridae (*Genetta tigrina*), with selection of these clades based on the overall topology in Nyakatura & Bininda-Emonds^67^ to obtain close relatives of Malagasy carnivorans. The consensus tree is provided in Supplementary Fig. S1. For primates, we used a recent inference of lemur phylogeny^68^, with a block of 200 posterior trees to account for phylogenetic uncertainty and comparison groups that included all other Strepsirrhini for which data were available, along with species in the genera *Tarsius*, *Saimiri*, *Aotus*, *Alouatta*, *Allenopithecus*, *Pan*, and *Homo* (consensus tree in Supplementary Fig. S2). For tenrecs, we used a block of 330 trees from Everson *et al*.^69^, which provided comparisons to five non-Malagasy non-tenrec species: *Amblysomus hottentotus*, *Chrysochloris stuhlmanni*, *Elephas maximus*, *Procavia capensis*, and *Petrodromus tetradactylus* (consensus tree in Supplementary Fig. S3). For rodents, we used a block of 200 trees from Schenk *et al*.^70^, with comparison groups that included 88 rodent species (consensus tree in Supplementary Fig. S4). Finally, for the global analysis of all mammals, we used the “best dates” mammal phylogeny of Fritz *et al*.^60^. All data are available as Supplementary Materials (Supplementary Table S3).

To quantify SSD for each species, we calculated an index based on the two-step ratio recommended by Smith^71^. This is the best possible ratio on a linear scale for data sets in which either males or females can be larger. This index is symmetrical around 1, with values >1 if males are the larger sex and <1 if females are the larger sex. In species with larger males, this index is calculated as the ratio of male and female mass and in cases where females are larger on average, as 2 − (female mass/male mass).

### Characterization of sexual size dimorphism

We compared log 10-transformed species means of female and male body mass and the corresponding SSD in non-lemur species for which new data on intraspecific variation in body mass were available to characterize the magnitude of sex differences in body mass within each species. For this, we conducted a meta-analysis using the “metafor” package^72^. In this analysis, each species was treated as a separate effect size measuring the difference between male and female body mass and associated standard error around this effect size. The goal was to infer the overall effect size of “sex” on body mass among mammals in Madagascar. We focused on standardized mean difference as the effect size, with positive values indicating male-biased sexual dimorphism. An overall effect size with 95% confidence intervals that bracket zero is consistent with a lack of sexual dimorphism, based on the overall null hypothesis that the sexes are generally equal in body mass in these lineages of Malagasy mammals. We provide results graphically in the form of a forest plot, which depicts the 95% confidence intervals for each of the species and enables readers to identify any species that depart from the overall patterns that we documented.

We first built a random effects model in metafor to investigate all species for which data were available in our database. In this model, the observed effects are assumed to be unbiased and normally distributed estimates of the true effect size – i.e., degree of dimorphism among Malagasy mammals in our sample – with known sampling variances and heterogeneity among different species. This analysis did not account for phylogeny, but does provide a larger sample size, as many of the species in our dataset could not be easily linked to the mammal phylogeny that is available^60^.

Following analysis of the full set of species, we then constructed a phylogenetic meta-analysis by restricting the analysis to those species that could be placed on the phylogeny used in the cross-mammals analysis^60^. We also estimated phylogenetic signal in effect sizes of dimorphism for this subset of species using the “caper” package^73^, specifically through maximum likelihood estimation of λ when setting dimorphism to a constant in the statistical model. We used this estimate of λ to transform the tree into a variance-covariance matrix that represents the expected non-independence in effect sizes based on the phylogeny and degree of phylogenetic signal. We then compared three meta-analytical models using AIC corrected for small samples (AICc). One of these analyses included no control for phylogeny, a second model used the untransformed variance-covariance matrix (i.e., based on branch lengths given in Fritz *et al*.^60^), and a final model used the variance-covariance matrix that was λ-transformed according to the degree of phylogenetic signal. We used default settings for model fit in the metafor functions rma.uni and rma.mv (i.e., restricted maximum likelihood).

### Evolutionary dynamics of sexual size dimorphism

We investigated evolutionary dynamics of SSD in each of the four endemic taxa of Malagasy mammals. In all cases, we are essentially comparing one clade nested within another larger clade, i.e. with a single evolutionary origin of the trait of interest on just one branch leading to the nested clade (in this case, colonization of Madagascar). Comparisons of this sort can have elevated Type I error rates unless appropriate phylogenetic controls are used^74^. Thus, we used two phylogeny-based methods to investigate change along the “colonizing” branches leading to Malagasy and non-Malagasy lineages, repeated separately for each of these sets of phylogenetic comparisons.

Our first method was based on phylogenetic generalized least squares (PGLS). This statistical model included “location” scored as a binary trait (0 = non-Malagasy, 1 = Malagasy). In the case of SSD, for example, the model would be represented as “SSD = *β*_*location*_ * location.” We thus refer to *β*_*location*_ throughout, i.e. the effect of location on the trait of interest (SSD, male or female body mass). We also ran tests of the effects of location on SSD in which we included female body mass as a predictor, i.e., SSD = *β*_*location*_ * location + *β*_*mass*_ * female body mass.

We used a Bayesian framework – implemented in R in BayesModelS^75^ – for statistical inference. This approach uses Markov Chain Monte Carlo (MCMC) to produce a posterior probability distribution of the regression coefficient (*β*_*location*_), along with Bayesian model selection to assess the probability that *β*_*location*_ should be included in the model. The details of this procedure are given in Nunn & Zhu^75^, and involve updating a vector that includes or excludes particular variables at steps in the Markov chain, and estimating those that are included. When *β*_*location*_ is included often in the model and is typically negative, this indicates that the Malagasy lineage shows lower values of the phenotypic trait in question.

We also estimated Pagel’s λ and Pagel’s κ in BayesModelS as scaling parameters to better meet the underlying assumptions of phenotypic evolution on the tree^74^. The parameter λ^76^ multiplies the internal branch lengths by a number from 0 to 1, with 0 equivalent to a star phylogeny and, thus, indicative of no phylogenetic signal. The parameter κ raises branch lengths to the value κ^77^. The κ parameter has previously been interpreted as in indicator of the “speciational” mode of evolution^78^; here, however, we include it to better meet the assumptions of Brownian motion that underlie the PGLS model. We invoked the option in BayesModelS to select whether to estimate λ or κ, thus using a model selection routine similar to that used for deciding whether to include *β*_*location*_ in the statistical model. We estimated these scaling parameters to improve overall fit to a Brownian motion model of evolution, rather than to make inferences about the tempo and mode of evolution.

Obtaining an effective MCMC chain requires settings to ensure that the samples of *β*_*location*_ and other parameters are sampled effectively, and without an overly high correlation between samples. Based on initial analyses and diagnostic tests of the output, we used the following settings. We ran analyses with a burnin of 100 iterations and sampled the MCMC chain every 50 iterations (thin rate), producing a posterior probability distribution of 1000 samples for estimating *β*_*location*_, probability of including *β*_*location*_ in the statistical model (model selection), and other parameters. To ensure adequate burnin and thin rate (i.e. sampling from a stable distribution of likelihoods with low correlation across neighboring samples), we checked that a plot of likelihoods had stabilized and showed low autocorrelation, and we used a flat prior for parameters in all analyses. Flat priors were used for all estimated coefficients in these models.

We specifically expected *β*_*location*_ would be included for SSD with negative regression coefficients. In addition to an effect size (regression coefficient), BayesModelS provides two sources of information for assessing the effect of Madagascar on SSD. First, it provides a probability that *β*_*location*_ should be included in the model. Second, among models in which *β*_*location*_ is included, it provides an estimate of the coefficient and the probability that the effect is negative (as predicted by the eco-evo-devo hypothesis). Barbiery and Berger^79^ provide mathematical support for including any parameters with more than 50% support in a maximally predictive model; though parameters with less support can still be meaningful, they are more difficult to interpret. Thus, we present both metrics, and avoid providing specific support levels, as it is unclear what cutoffs should be used for interpreting these posterior probabilities.

To conduct the analysis of all mammals, we used a different procedure because the sample size of the analysis (n = 1379 species) exceeded the capability of BayesModelS and we lacked a Bayesian posterior distribution of phylogenies. For this analysis, we used the R package caper^72^ to assess correlated evolution between SSD, female body mass, male body mass, and residence on Madagascar. We compared the AIC of models with and without relevant predictor variables, and present regression coefficients and R^2^ values. The phylogeny of Fritz *et al*.^60^ was incomplete with regard to current taxonomic assessments of Malagasy mammals, resulting in 48 species of mammals representing each of the Malagasy lineages examined in the clade-by-clade analyses. Data used for this analysis are presented in Table S2 in Supplementary Materials.

In addition to these PGLS methods, we applied an Ornstein-Uhlenbeck (OU) model of adaptive change that allows multiple adaptive regimes across a phylogeny^59^. This Bayesian approach estimates the probability of regime shifts throughout the phylogeny; from the analysis, it is possible to identify branches with the highest probability of shifts, and to identify those inferred shifts as increases or decreases in the adaptive optimum. Lineages may have the same or different adaptive regimes. The goal is to characterize the regimes across the tree, and to assess whether the regime has undergone a shift along the branches leading to the Malagasy lineages. We focus on support for regime shifts on the branch leading to Madagascar. To visualize these inferred changes across the phylogeny, we plotted regime shifts on branches only when 10% or more of the MCMC samples inferred such a change. As with BayesModelS, it is unclear what probability level should be used as evidence that a shift in the adaptive regime occurred. We note, however, that with the large number of potential models that attempt to fit different adaptive regimes across all branch lengths, even 10% support probabilities are likely providing meaningful signal of shifts along particular branches. When relevant, we provide probability levels for evolutionary changes on other branches to enable comparison of support across the tree.

To implement this approach, we used the R package bayou^80^, which is a Bayesian implementation of OU model-fitting. This method addresses many statistical concerns with OU models of evolution, including issues with model selection^81^ and estimates of where evolutionary changes occur on the tree^82^. However, it tends to produce more auto-correlated MCMCs and has a more complex underlying model than our other method; thus, to achieve large effective sample sizes, we ran much longer chains and collected a larger posterior distribution than we did with BayesModelS. We also ran two chains and ensured convergence by graphical inspection of the output and by comparing the magnitude of shifts across branches in the two runs, aiming for strong correlations between estimated parameters across runs. We then used and plotted the Gelman-Rubin convergence diagnostic to visually confirm that it stabilized near one and removed the portion of each chain preceding this point of convergence. We attempted to use bayou to analyze the full mammal sample of 1379 species but were unable to get the chains to converge.

## Electronic supplementary material

Dataset 1

## Data Availability

All raw data are available as supplementary electronic material associated with this article.
