# Supplementary material for: Evolutionary dynamics of sexual size dimorphism in non-volant mammals following their independent colonization of Madagascar
Source: Sci Rep. 2019 Feb 5;9:1454. doi: 10.1038/s41598-018-36246-x (PMC6363729; doi:10.1038/s41598-018-36246-x)
Supplement: Supplementary file 1 — Dataset 1 [file 41598_2018_36246_MOESM1_ESM.pdf]

Evolutionary dynamics of sexual size dimorphism in non-volant mammals following their independent colonization of Madagascar

Peter M. Kappeler, Charles L. Nunn, Alexander Q. Vining & Steven M. Goodman

Supplementary Electronic Material

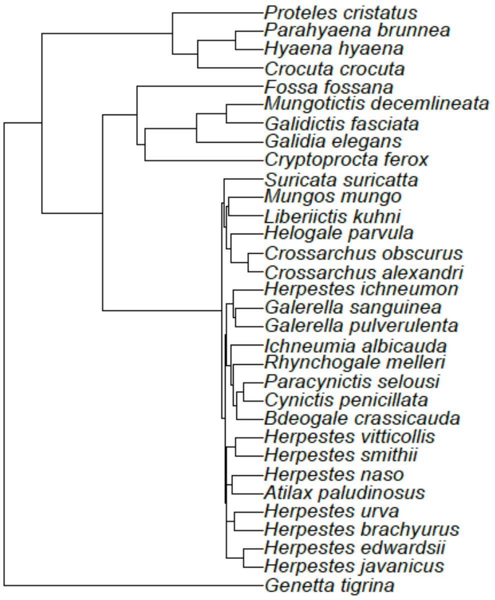

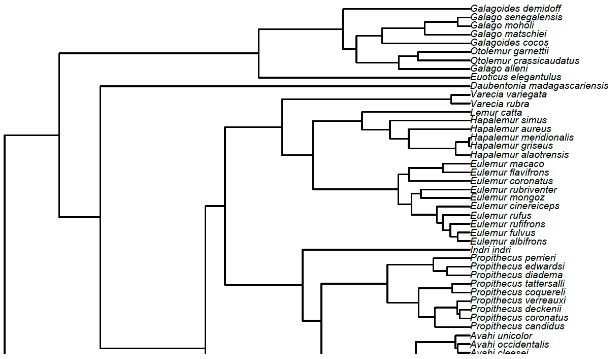

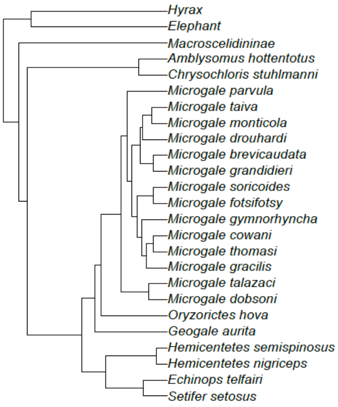

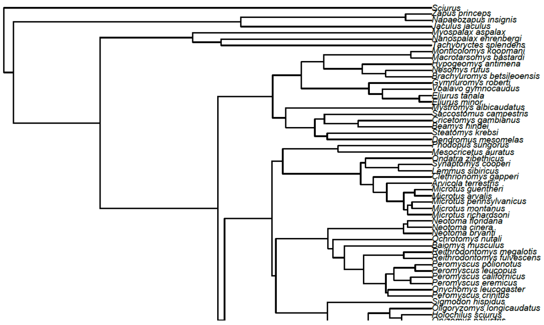

Figurs

S1-S4 Phylogenies used in comparative analyses for carnivorans, primates, tenrecs (Afrotheria), and rodents, respectively. The full mammal tree is from a different source, and not presented visually because of its size.

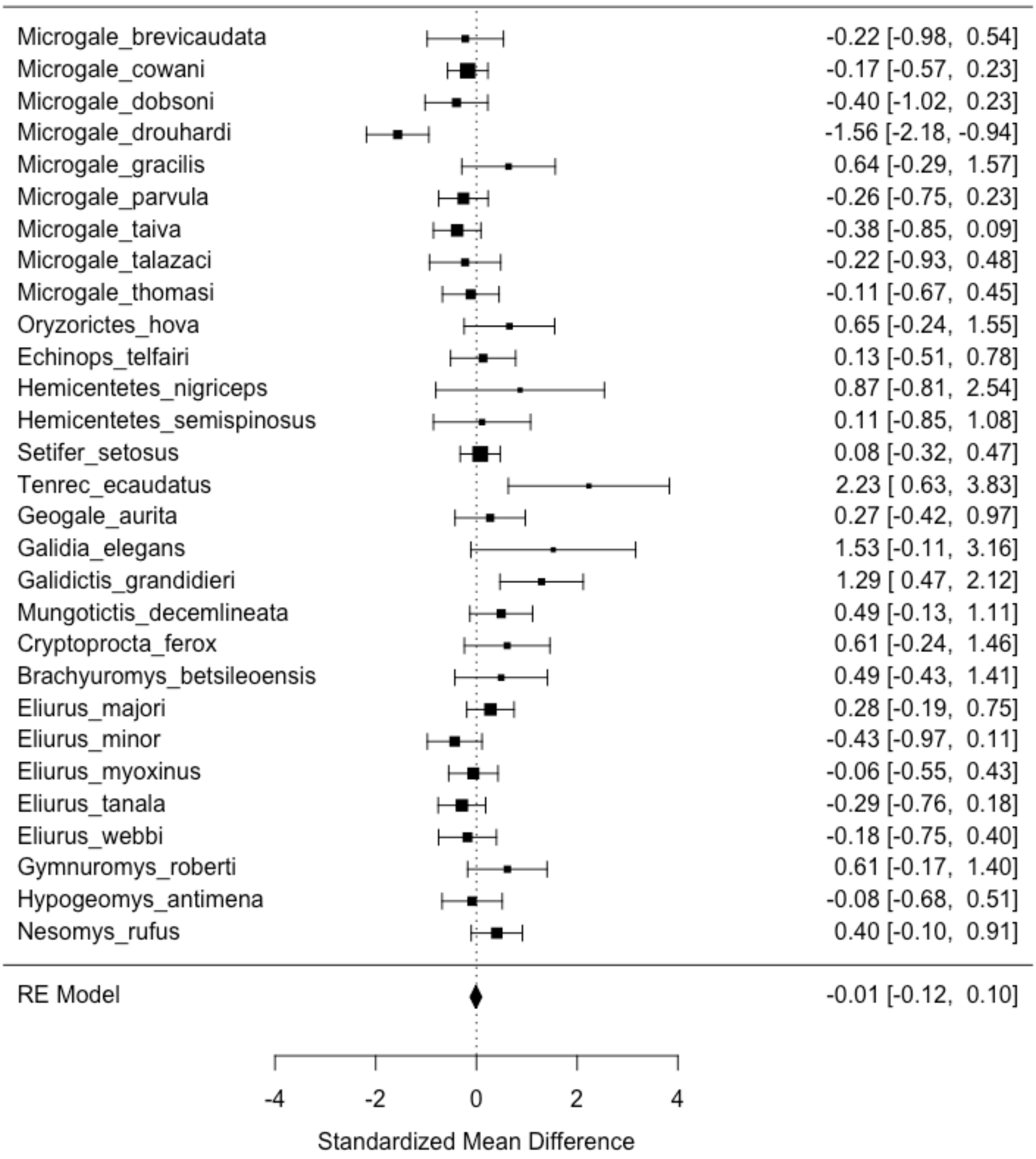

Figure S5 Patterns of SSD in non-primate Malagasy mammals. Depicted are results of a meta-analysis treating each species as a separate study, with the goal to infer the overall effect size of "sex" on body mass. Effect sizes are represented as standardized mean difference, with positive values indicating male-biased sexual dimorphism, and their 95% confidence intervals. This figure includes only species for which phylogenetic information was available (cf. Fig. 2).

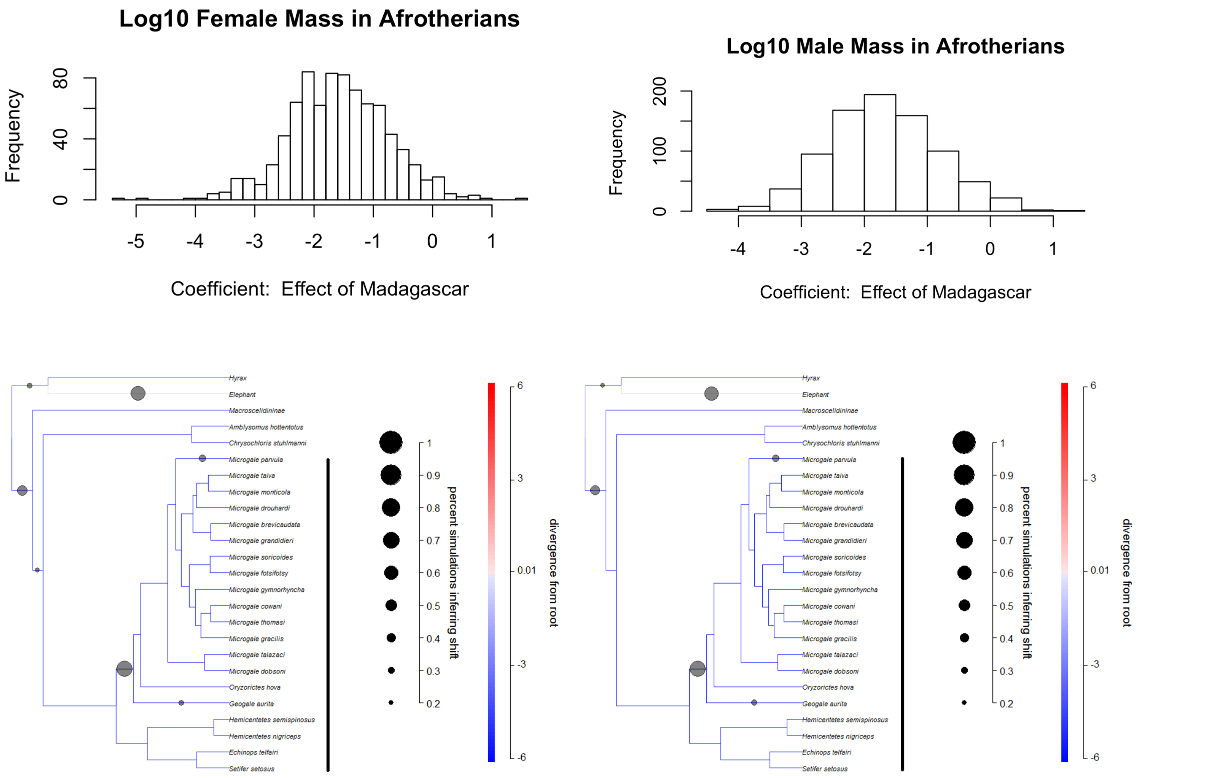

Figure S6 Female and male body mass evolution in tenrecs (Afrotheria). PGLS revealed some evidence for decreases in (a) female and (b) male mass, with most posterior distributions of estimated coefficients fairly well centered below zero. Bayou analyses revealed larger changes in (c) female and (d) male body mass in sub-clades of the tree, as compared to the basal lineages.

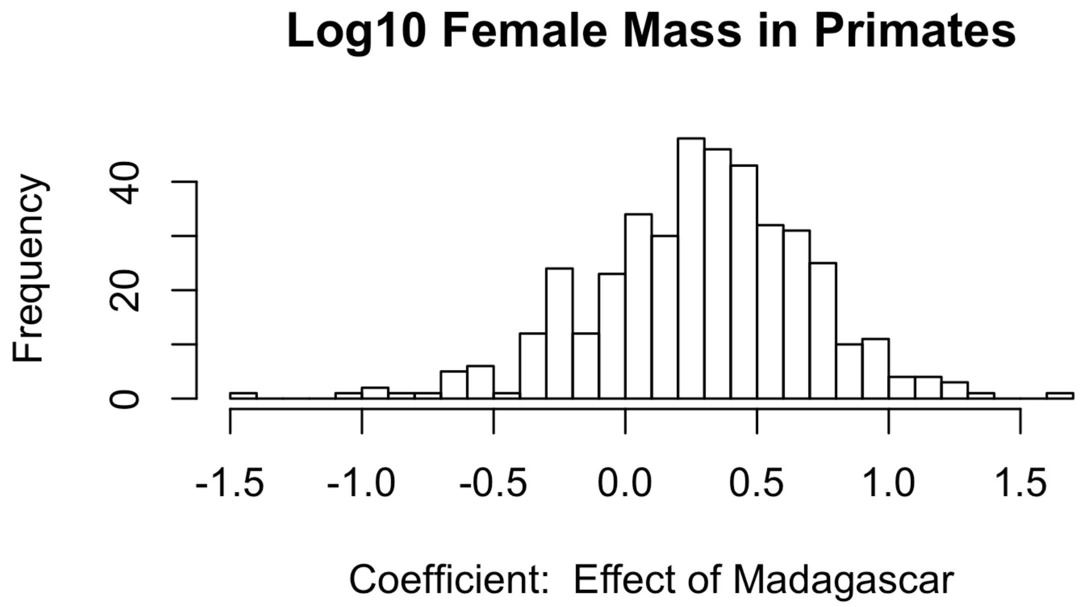

Fig. S7 Female body mass evolution in primates. The decline in Malagasy primate SSD appears driven in part by increases in female body mass, with most estimated regression coefficients in the posterior probability distribution being positive.

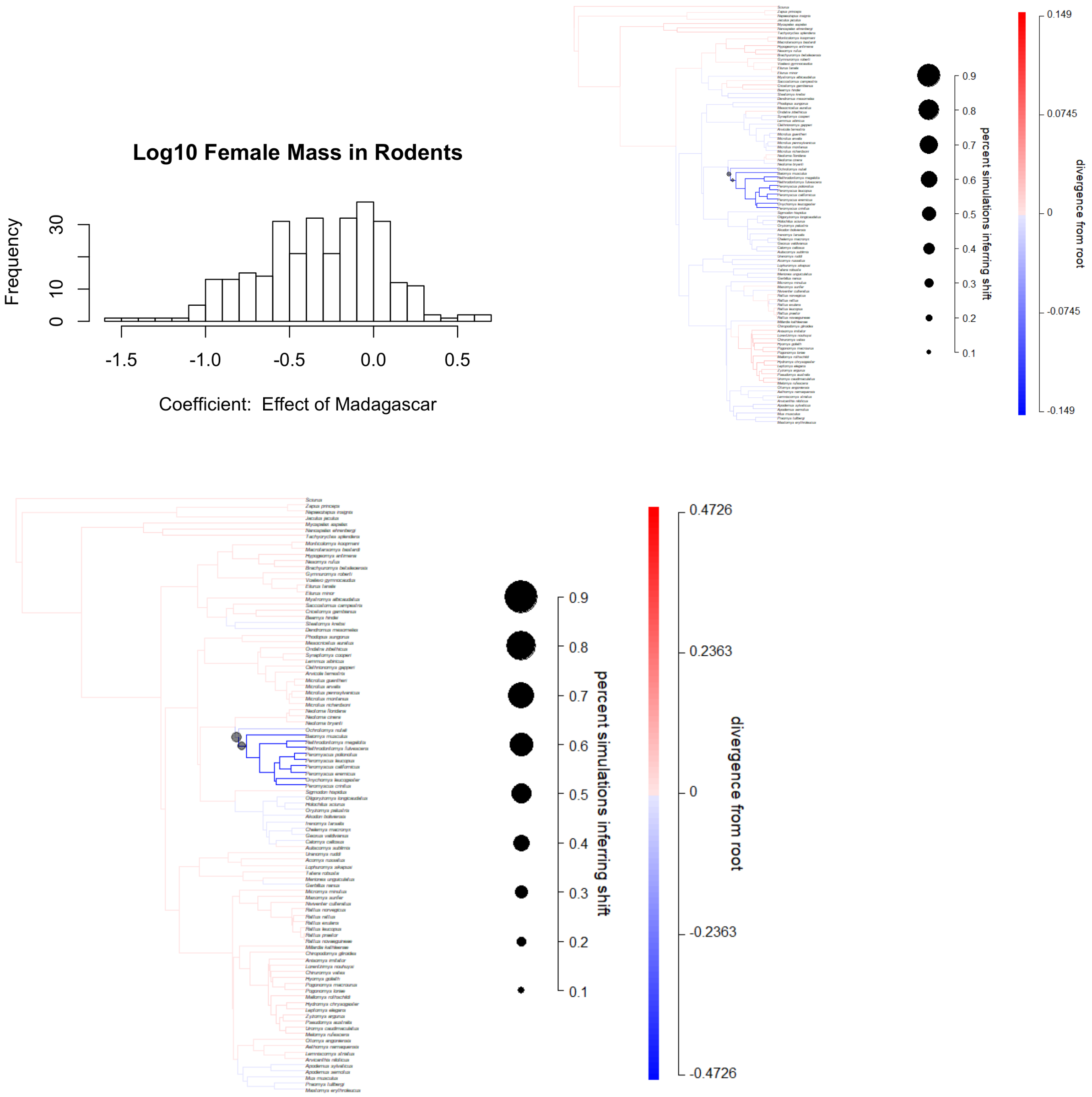

Fig. S8 Female body mass evolution in rodents. (a) There was no support for changes in female body mass in Malagasy rodents based on a PGLS model in BayesModelS. Bayou revealed no support for changes in female (b) or male (c) body mass in Malagasy rodents.

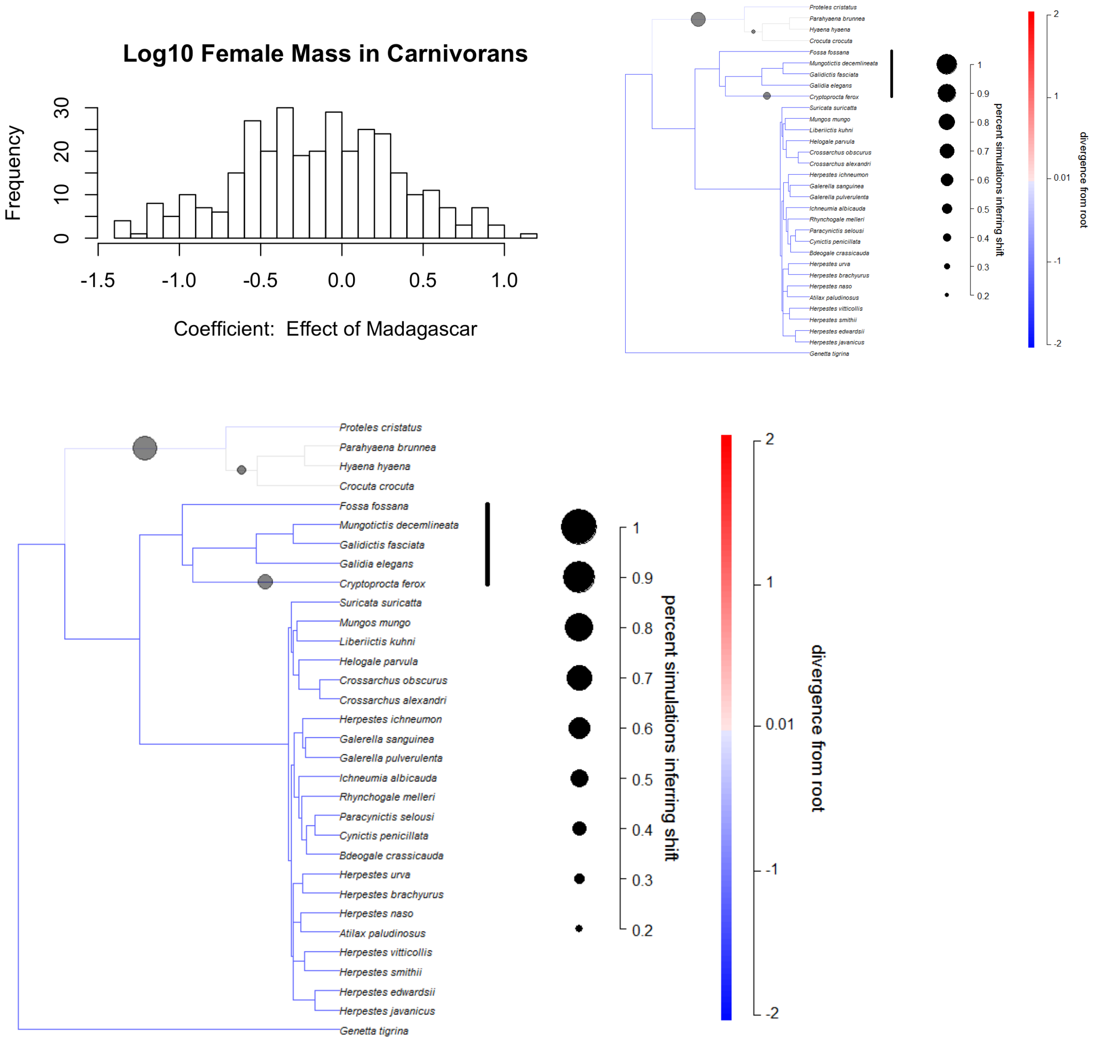

Fig. S9 Body mass evolution in Carnivorans. Phylogenetic analyses revealed little support for changes in female body mass in Malagasy lineages, based on phylogenetic PGLS in BayesModelS and (a) reconstructed regime changes for both sexes (b, c) in bayou.

Table S1: Body mass and sexual dimorphism in Malagasy primates (Lemuriformes)

Species	F	N	M	N	SSD	Ref
**Cheirogaleidae**						
Allocebus trichotis	76.0	9	79.0	5	1.04	Taylor and Schwitzer 2012
Cheirogaleus major	443.0	3	575.0	3	1.30	Rowe and Myers 2011
Cheirogaleus medius	143.0	36	132.0	63	0.92	Taylor and Schwitzer 2012
Microcebus berthae	31.0	54	31.0	108	1.00	Taylor and Schwitzer 2012
Microcebus gerpi	69.0	3	67.0	4	0.97	Taylor and Schwitzer 2012
Microcebus griseorufus	53.0	57	47.0	69	0.97	Taylor and Schwitzer 2012
Microcebus margotmarshae	52.0	3	42.0	1	0.87	Taylor and Schwitzer 2012
Microcebus murinus	63.0	168	59.0	76	0.76	Taylor and Schwitzer 2012
Microcebus ravelobensis	55.0	17	60.0	7	1.09	Taylor and Schwitzer 2012
Microcebus tavaratra	51.0	10	53.0	10	1.04	Taylor and Schwitzer 2012
Mirza coquereli	299.0	26	317.0	30	1.06	Taylor and Schwitzer 2012
Mirza zaza	295.0	10	286.0	16	0.97	Taylor and Schwitzer 2012
Phaner pallescens	351.0	6	328.0	8	0.93	Rowe and Myers 2011
**Daubentoniidae**						
Daubentonia madagascariensis	2446.0	15	2621.0	13	1.07	Taylor and Schwitzer 2012
**Indriidae**						
Avahi betsileo	1225.0	4	987.0	6	0.76	Taylor and Schwitzer 2012
Avahi cleesi	975.0	5	926.0	7	0.95	Taylor and Schwitzer 2012
Avahi laniger	1282.0	22	1068.0	20	0.80	Taylor and Schwitzer 2012
Avahi meridionalis	1130.0	10	1043.0	7	0.92	Taylor and Schwitzer 2012
Avahi mooreorum	950.0	3	904.0	4	0.95	Taylor and Schwitzer 2012
Avahi occidentalis	967.0	14	842.0	18	0.85	Taylor and Schwitzer 2012
Avahi peyrierasi	1120.0	13	990.0	25	0.87	Taylor and Schwitzer 2012
Avahi ramanantsoavanai	1031.0	8	941.0	9	0.90	Taylor and Schwitzer 2012
Avahi unicolor	920.0	1	830.0	3	0.89	Taylor and Schwitzer 2012
Indri indri	7135.0	2	5825.0	2	0.78	Taylor and Schwitzer 2012
Propithecus candidus	6000.0	1	5033.0	3	0.81	Taylor and Schwitzer 2012
Propithecus coquereli	3700.0	9	3700.0	16	1.00	Taylor and Schwitzer 2012
Propithecus coronatus	3738.0	5	3206.0	7	0.83	Taylor and Schwitzer 2012
Propithecus deckenii	2630.0	10	2930.0	10	1.11	Taylor and Schwitzer 2012
Propithecus diadema	6750.0	4	6496.0	5	0.96	Taylor and Schwitzer 2012
Propithecus edwardsi	5504.0	26	5500.0	30	1.00	Taylor and Schwitzer 2012
Propithecus perrieri	4519.0	21	4436.0	14	0.98	Taylor and Schwitzer 2012
Propithecus	3590.0	10	3390.0	8	0.94	Rowe and Myers 2011

tattersalli						
Propithecus verreauxi	2850.0	132	2890.0	165	1.01	Taylor and Schwitzer 2012
**Lemuridae**						
Eulemur albifrons	2150.0		2213.0		1.03	Rowe and Myers 2011
Eulemur cinereiceps	1995.0	26	1923.0	24	0.96	Taylor and Schwitzer 2012
Eulemur coronatus	1080.0	2	1280.0	2	1.19	Taylor and Schwitzer 2012
Eulemur flavifrons	2042.0	3	1797.0	8	0.86	Taylor and Schwitzer 2012
Eulemur fulvus	1500.0	7	1600.0	3	1.07	Taylor and Schwitzer 2012
Eulemur macaco	1987.0	28	1974.0	26	0.99	Taylor and Schwitzer 2012
Eulemur mongoz	1280.0	13	1140.0	9	0.88	Taylor and Schwitzer 2012
Eulemur rubriventer	1964.0	13	2067.0	9	1.05	Taylor and Schwitzer 2012
Eulemur rufifrons	2232.0	36	2096.0	53	0.94	Taylor and Schwitzer 2012
Eulemur rufus	1260.0	7	1410.0	13	1.12	Taylor and Schwitzer 2012
Hapalemur alaotrensis	1262.0	28	1235.0	33	0.98	Taylor and Schwitzer 2012
Hapalemur aureus	1500.0	1	1620.0	2	1.08	Taylor and Schwitzer 2012
Hapalemur griseus	838.0	8	838.0	9	1.00	Taylor and Schwitzer 2012
Hapalemur meridionalis	870.0	6	839.0	6	0.96	Taylor and Schwitzer 2012
Lemur catta	2268.0	89	2227.0	100	0.98	Taylor and Schwitzer 2012
Prolemur simus	2248.0	10	2532.0	10	1.13	Taylor and Schwitzer 2012
Varecia rubra	3640.0	2	3300.0	1	0.90	Taylor and Schwitzer 2012
Varecia variegata	3748.0	41	3624.0	49	0.97	Taylor and Schwitzer 2012
**Lepilemuridae**						
Lepilemur aeeclis	920.0	6	832.0	8	0.89	Taylor and Schwitzer 2012
Lepilemur ahmansonorum	500.0	2	650.0	1	1.30	Taylor and Schwitzer 2012
Lepilemur ankaranensis	779.0	12	733.0	9	0.94	Taylor and Schwitzer 2012
Lepilemur betsileo	1210.0	1	1000.0	1	0.79	Taylor and Schwitzer 2012
Lepilemur dorsalis	730.0	2	730.0	3	1.00	Taylor and Schwitzer 2012
Lepilemur edwardsi	983.0	19	982.0	17	1.00	Taylor and Schwitzer 2012
Lepilemur grewcockorum	725.0	2	900.0	1	1.24	Taylor and Schwitzer 2012
Lepilemur hollandorum	983.0	3	1000.0	2	1.02	Taylor and Schwitzer 2012
Lepilemur hubbardorum	837.0	3	1113.0	4	1.33	Taylor and Schwitzer 2012
Lepilemur jamesorum	1050.0	2	930.0	5	0.87	Taylor and Schwitzer 2012
Lepilemur leucopus	561.0	15	543.0	10	0.97	Taylor and Schwitzer 2012
Lepilemur microdon	872.0	5	1111.0	8	1.27	Taylor and Schwitzer 2012
Lepilemur milanoii	702.0	5	718.0	6	1.02	Taylor and Schwitzer 2012
Lepilemur mustelinus	1043.0	18	957.0	21	0.91	Taylor and Schwitzer 2012
Lepilemur petteri	625.0	4	640.0	1	1.02	Taylor and Schwitzer 2012
Lepilemur randrianasoli	804.0	5	748.0	6	0.93	Taylor and Schwitzer 2012
Lepilemur	758.0	32	752.0	27	0.99	Taylor and Schwitzer 2012

ruficaudatus						
Lepilemur sahamalazensis	759.0	10	701.0	4	0.92	Taylor and Schwitzer 2012
Lepilemur seali	950.0	2	957.0	3	1.01	Taylor and Schwitzer 2012
Lepilemur tymerlachsonorum	893.0	14	825.0	12	0.92	Taylor and Schwitzer 2012
Lepilemur wrightae	1217.0	3	1050.0	2	0.84	Taylor and Schwitzer 2012

Mean adult body mass of adult females (F) and males (M) along with the respective standard deviation(SD) and sample size (N). Sexual size dimorphism (SSD) is expressed as the two-step ratio.

References:

Rowe, N. & Myers, M. 2011. *All the World's Primates*. Primate Conservation Inc., Charlestown, RI.

Taylor, L.A. & Schwitzer, C. 2012. Body masses of wild lemurs. *Lemur News* **16**: 34–40.

Table S2: Body mass and sexual dimorphism in sister clades or ecological equivalents of Malagasy mammals used in direct comparisons

Subfamily	Species	F	N	M	N	SSD	Reference
**Nesomyidae**							
Mystromyinae	*Mystromys albicauda*	82.9	53	110.0	51	1.33	Becker and Middleton 1979
Cricetomyinae	*Beamys hindei*	67.0	10	69.0	5	1.03	Happold 2013
	Cricetomys emini	902.7	39	935.5	22	1.04	Happold 2013
	Cricetomys gambianus	1140.0	171	1280.0	192	1.12	Dzenda et al. 2011
	Saccostomus campestris	51.0	19	63.0	13	1.24	Ellison et al. 1993
	Saccostomus mearnsi	62.0	97	79.0	121	1.27	Happold 2013
Dendromurinae	*Dendromus melanotis*	7.0	19	7.4	11	1.06	Happold 2013
	Dendromus mesomelas	10.6	5	12.0	4	1.13	Happold 2013
	Dendromus mystacalis	8.7	3	7.2	6	0.79	Happold 2013
	Steatomys krebsi	18.0	3	20.0	3	1.11	Skinner and Chimimba 2005
	Steatomys pratensis	28.5	20	28.3	23	0.99	Monadjem 1999
**Chrysochloridae**							
Chrysochlorinae	*Carpitalpa arendsi*	41.6	11	52.7	11	1.26	Skinner and Chimimba 2005; Kingdon et al. 2013
	Chlorotalpa duthiae	26.0	3	33.0	3	1.27	Kingdon et al. 2013
	Chlorotalpa sclateri	33.7	11	39.5	15	1.17	Skinner and Chimimba 2005
	Chrysospalax trevelyani	455.0	2	470.0	1	1.03	Kingdon et al. 2013
	Chrysospalax villosus	99.0	2	137.7	3	1.39	Kingdon et al. 2013
	Eremitalpa granti	19.8	23	25.3	17	1.28	Skinner and Chimimba 2005
Ambylosominae	*Amblysomus corriae*	47	13	56	10	1.19	Kingdon et al. 2013
	Amblysomus hottentotus	50.0	28	55.6	28	1.13	Skinner and Chimimba 2005; Kingdon et al. 2013
	Amblysomus robustus	67.0	2	85.0	2	1.27	Kingdon et al. 2013
	Calcochloris obtusirostris	24.5	11	28.1	9	1.15	Kingdon et al. 2013
	Neamblysomus gunningi	51.0	4	62.8	6	1.23	Kingdon et al. 2013
	Neamblysomus julianae	26.0	5	30.0	5	1.15	Kingdon et al. 2013
**Herpestidae**							

Herpestinae	*Atilax paludinosus*	2600.0	19	3000.0	18	1.15	Kingdon and Hoffmann 2013
	Bdeogale crassicaudata	1479.3	6	1601	6	1.08	Martinoli et al. 2006; Kingdon and Hoffmann 2013
	Cynictis penicillata	808.0	5	900	6	1.11	Cavallini 1993
	Galerella nigrata	850	1	821	5	0.96	Rathburn and Cowley 2008
	Galerella sanguinea	567.6	13	695.4	16	1.23	Waser et al. 1995; Waser unpubl.
	Herpestes ichneumon	3163.6	11	3330.8	13	1.05	Skinner and Chimimba 2005; Kingdon and Hoffmann 2013
	Herpestes pulverentulus	683.0	28	911.0	39	1.33	Kingdon and Hoffmann 2013
	Ichneumia albicauda	3521.2	17	3575.7	14	1.02	Waser and Waser 1985; Waser unpubl.
	Paracynictis selousi	1750.0	21	1750.0	18	1.00	Kingdon and Hoffmann 2013
	Xenogale naso	2533.3	15	2980.0	10	1.18	Ray 1997; Kingdon and Hoffmann 2013
Mungotinae	*Crossarchus alexandri*	1450	1	1470.0	2	1.01	Kingdon and Hoffmann 2013
	Helogale parvula	331.1	57	339.4	61	1.03	Waser et al. 1985; Waser unpubl.
	Mungos mungo	1428.5	61	1478.8	133	1.04	Kingdon and Hoffmann 2013; Waser unpubl.
	Suricata suricatta	639.7	483	652.8	512	1.02	Huchard and Clutton-Brock unpubl.
**Galagidae**							
Galaginae	*Euoticus elegantulus*	261.0	3	287.0	5	1.10	Smith and Jungers 1997
	Euticus pallidus	241.0	2	279	1	1.16	Butynski et al. 2013
	Galago matschiei	212.0	4	207.0	2	0.98	Smith and Jungers 1997
	Galago moholi	173.0	81	187.0	112	1.08	Smith and Jungers 1997
	Galago senegalensis	199.0	67	227.0	80	1.14	Smith and Jungers 1997
	Galagoides cocos	138.0	42	150.0	36	1.09	Butynski et al. 2013
	Galagoides demidovii	55.0	16	60.0	17	1.09	Butynski et al. 2013
	Galagoides thomasi	75.0	6	82.0	6	1.09	Butynski et al. 2013
	Otolemur crassicaudatus	1130.0	13	1220.0	24	1.08	Butynski et al. 2013
	Otolemur garnetti	794.3	14	861.5	18	1.08	Butynski et al. 2013
	Sciurocheirus alleni	269.0	30	277.0	9	1.03	Smith and Jungers 1997
**Soricidae**							
	Crocidura allex	4.66	46	5.24	100	1.12	Stanley et al. 2015; Stanley pers. comm.
	Crocidura crossei	5.1	4	5.6	5	1.10	Happold and Happold 2013

	Crocidura fischeri	13.1	4	14.8	3	1.13	Happold and Happold 2013
	Crocidura flavescens	25.2	17	30.0	10	1.19	Happold and Happold 2013
	Crocidura fulvastra	14.0	5	22.0	4	1.57	Happold and Happold 2013
	Crocidura jacksoni	8.1	9	8.5	16	1.05	Happold and Happold 2013
	Crocidura mariquensis	9.0	50	11.7	46	1.30	Happold and Happold 2013
	Crocidura maurisca	9.2	4	13.0	14	1.41	Happold and Happold 2013
	Crocidura mdumai	8.3	6	8.7	12	1.05	Stanley et al. 2015; Stanley pers. comm.
	Crocidura monax	13.9	19	14.2	53	1.02	Stanley et al. 2015; Stanley pers. comm.
	Crocidura newmarki	8.3	52	8.7	83	1.04	Stanley et al. 2015; Stanley pers. comm.
	Crocidura nigeriae	15.3	4	20.3	3	1.33	Happold and Happold 2013
	Crocidura olivieri	33.8	4	35.4	3	1.05	Happold and Happold 2013
	Crocidura usambarae	8.7	2	9.7	6	1.11	Happold and Happold 2013
	Myosorex longicaudatus	9.5	2	14	72	1.47	Happold and Happold 2013
	Suncus aequatorius	8.7	2	8.9	10	1.02	Happold and Happold 2013
	Suncus murinus	67.0		105.0		1.57	Happold and Happold 2013
	Sylvisorex granti	5.7	24	6.0	35	1.05	Stanley et al. 2015; Stanley pers. comm.
	Sylvisorex howelli	3.8	82	4.2	138	1.12	Stanley et al. 2015; Stanley pers. comm.

Mean adult body mass of adult females (F) and males (M) along with the respective standard deviation and sample size. Sexual size dimorphism (SSD) is expressed as the two-step ratio. Most of these data were not included in Lindenfors et al. 2007.

References

Becker, S.V. & Middleton, C.C. 1979. Organ weights and organ: body weight ratios of the African white-tailed rat (*Mystromys albicaudatus*). *Lab. Anim. Sci.* **29**: 44–47.

Butynski, T.M., Kingdon, J. & Kalina, J. 2013. *Mammals of Africa. Volume II: Primates*. Bloomsbury Publishing, London.

Cavallini, P. 1993. Spatial organization of the yellow mongoose *Cynictis penicillata* in a coastal area. *Ethol. Ecol. Evol.* **5**: 501–509.

Dzenda, T., Ayo, J.O., Lakpini, C.A.M. & Adelaiye, A.B. 2011. Seasonal and sex variations in live weights of captive African giant rats (*Cricetomys gambianus* Waterhouse) in the Northern Guinea savannah zone of Nigeria. *Int. J. Zool. Res.* **7**: 49–58.

Ellison, G.T.H., Taylor, P.J., Nix, H.A., Bronner, G.N. & McMahon, J.P. 1993. Climatic adaptation of body size among pouched mice (*Saccostomus campestris*: Cricetidae) in the Southern African subregion. *Global Ecol. Biogeogr. Lett.* **3**: 41–47.

Happold, D.C.D. 2013. *Mammals of Africa. Volume III: Rodents, Hares and Rabbits.* Bloomsbury Publishing, London.

Happold M, Happold DCD (2013) Mammals of Africa. Vol IV: Hedgehogs, Shrews and Bats. Bloomsbury Publishing, London.

Kingdon, J. & Hoffman, M. 2013. *Mammals of Africa. Volume V: Carnivores, Pangolins, Equids and Rhinoceroses*. Bloomsbury Publishing, London.

Kingdon, J., Happold, D.C.D., Hoffmann, M., Butynski, T.M., Happold, M. & Kalina, J. 2013. *Mammals of Africa. Volume I: Introductory Chapters and Afrotheria*. Bloomsbury Publishing, London.

Martinoli, A., Preatoni, D., Galanti, V., Codipietro, P., Kilewo, M., Fernandes, C.R. *et al.* 2006. Species richness and habitat use of small carnivores in the Arusha National Park (Tanzania). *Biodivers. Conserv.* **15**: 1729–1744.

Monadjem, A. 1999. Population dynamics of *Mus minutoides* and *Steatomys pratensis* (Muridae: Rodentia) in a subtropical grassland in Swaziland. *Afr. J. Ecol.* **37**: 202–210.

Rathbun, G.B. & Cowley, T.E. 2008. Behavioural ecology of the black mongoose (*Galerella nigrata*) in Namibia. *Mamm. Biol.* **73**: 444–450.

Ray, J.C. 1997. Comparative ecology of two African forest mongooses, *Herpestes naso* and *Atilax paludinosus*. *Afr. J. Ecol.* **35**: 237–253.

Skinner, J.D. & Chimimba, C.T. 2005. *The Mammals of the Southern African Sub-Region.* Cambridge University Press, Cambridge.

Smith, R.J. & Jungers, W.L. 1997. Body mass in comparative primatology. *J. Hum. Evol.* **32**: 523–559.

Stanley, W.T., Hutterer, R., Giarla, T.C. & Esselstyn, J.A. 2015. Phylogeny, phylogeography and geographical variation in the *Crocidura monax* (Soricidae) species complex from the montane islands of Tanzania, with descriptions of three new species. *Zool. J. Linn. Soc.* **174**: 185–215.

Waser, P.M. & Waser, M.S. 1985. *Ichneumia alhicauda* and the evolution of viverrid gregariousness. *Z. Tierpsychol.* **68**: 137–151.

Waser, P.M., Elliott, L.F., Creel, N.M. & Creel, S.R. 1995. Habitat variation and mongoose demography. In: *Serengeti II: Dynamics, Management and Conservation of an Ecosystem* (A.R.E. Sinclair & P. Arcese, eds), pp. 421–447. University of Chicago Press, Chicago.

Data used in analyses of four independent clades of Malagasy mammals

Tenrecs and their comparison group:

Species_Tree	*Species_Data*	*Source*	*Madagascar*	*female_mass*	*male_mass*	*SSD*	*log10_fem_m*	*log10_male_mass*
Amblysomus	Amblysomus	Goodman &	0	50	56.6	1.13	1.70	1.75
Chrysochlori	Chrysochlori	Pantheria	0	52.2	68.5	1.31	1.72	1.84
Elephant	Elephas_max	Pantheria, us	0	####	####	1.83	6.43	6.70
Hyrax	Procavia_cap	Pantheria	0	3600	4000	1.11	3.56	3.60
Macroscelidi	Petrodromus	Pantheria	0	220	215	0.98	2.34	2.33
Echinops_tel	Echinops_tel	Goodman &	1	99.9	102	1.03	2.00	2.01
Geogale_aur	Geogale_aur	Goodman &	1	7	7.3	1.05	0.85	0.87
Hemicentete	Hemicentete	Goodman &	1	98	111	1.13	1.99	2.05
Hemicentete	Hemicentete	Goodman &	1	108	110	1.02	2.03	2.04
Microgale_b	Microgale_b	Goodman &	1	9.7	9.4	0.96	0.99	0.97
Microgale_c	Microgale_c	Goodman &	1	12.7	12.4	0.97	1.10	1.09
Microgale_d	Microgale_d	Goodman &	1	28.9	26.9	0.93	1.46	1.43
Microgale_d	Microgale_d	Goodman &	1	12.5	10.1	0.76	1.10	1.00
Microgale_fo	Microgale_fo	Goodman &	1	9.2	7.8	0.83	0.96	0.89
Microgale_g	Microgale_g	Goodman &	1	22.2	23.9	1.08	1.35	1.38
Microgale_g	Microgale_g	Goodman &	1	8.3	9	1.08	0.92	0.96
Microgale_g	Microgale_g	Goodman &	1	17.7	17.6	0.99	1.25	1.25
Microgale_m	Microgale_m	Goodman &	1	14	12.8	0.91	1.15	1.11
Microgale_p	Microgale_p	Goodman &	1	3.2	3.1	0.96	0.51	0.49
Microgale_s	Microgale_s	Goodman &	1	19.3	18	0.93	1.29	1.25
Microgale_ta	Microgale_ta	Goodman &	1	12.3	11.7	0.95	1.09	1.07
Microgale_ta	Microgale_ta	Goodman &	1	39.7	38.9	0.98	1.60	1.59
Microgale_th	Microgale_th	Goodman &	1	22.1	21.7	0.98	1.34	1.34
Oryzorictes_	Oryzorictes_	Goodman &	1	33	37.2	1.13	1.52	1.57
Setifer_setos	Setifer_setos	Goodman &	1	219	223	1.02	2.34	2.35

Lemurs and their comparison group:

Species	*10KTrees*	*10kTrees_Ta*	*Herrara*	*Herrara_phy*	*Source*	*Madagascar*	*female_mass*	*log10_fem_m*	*male_mass*	*log10_male_*	*SSD*	
Euoticus_ele	1	Euoticus_ele	1	Euoticus_ele	this study	0	261	2.42	287	2.46	1.10	
Euoticus_pal	0	Euoticus_pal	0	Euoticus_pal	this study	0	241	2.38	279	2.45	1.16	
Galago_mats	1	Galago_mats	1	Galago_mats	this study	0	212	2.33	207	2.32	0.98	
Galago_moh	1	Galago_moh	1	Galago_moh	this study	0	173	2.24	187	2.27	1.08	
Galago_sene	1	Galago_sene	1	Galago_sene	this study	0	199	2.30	227	2.36	1.14	
Galagoides_	0	Galagoides_	1	Galagoides_	this study	0	138	2.14	150	2.18	1.09	
Galagoides_	1	Galago demi	1	Galagoides_	this study	0	55	1.74	60	1.78	1.09	
Galagoides_	0	Galagoides_	0	Galagoides_	this study	0	75	1.88	82	1.91	1.09	
Otolemur_cr	1	Otolemur_cr	1	Otolemur_cr	this study	0	1130	3.05	1220	3.09	1.08	
Otolemur_ga	1	Otolemur_ga	1	Otolemur_ga	this study	0	794	2.90	862	2.94	1.08	
Sciurocheiru	0	Sciurocheiru	1	Galago_allen	this study	0	269	2.43	277	2.44	1.03	
Tarsius_syric	NA	Tarsius_syrichta		Tarsius_syric	Pantheria	0	117	2.07	134	2.13	1.15	not used in Fig.1.
Tarsius_banc	NA	Tarsius_bancanus		Tarsius_banc	Pantheria	0	117	2.07	128	2.11	1.09	not used in Fig.1.
Saimiri_sciur	NA	Saimiri_sciureus		Saimiri_sciur	Pantheria	0	680	2.83	899	2.95	1.32	not used in Fig.1.
Aotus_trivirg	NA	Aotus_trivirgatus		Aotus_trivirg	Pantheria	0	736	2.87	813	2.91	1.10	not used in Fig.1.
Alouatta_sen	NA	Alouatta_seniculus		Alouatta_sen	Pantheria	0	5300	3.72	6873	3.84	1.30	not used in Fig.1.
Allenopithec	NA	Allenopithecus_nigrovirid		Allenopithec	Pantheria	0	3180	3.50	6130	3.79	1.93	not used in Fig.1.
Pan_troglody	NA	Pan_troglodytes		Pan_troglody	Pantheria	0	####	4.61	####	4.70	1.23	not used in Fig.1.
Homo_sapie	NA	Homo_sapiens		Homo_sapie	Pantheria	0	####	4.74	####	4.79	1.14	not used in Fig.1.
Allocebus_tr	1	Allocebus_tr	1	Allocebus_tr	this study	1	76	1.88	79	1.90	1.04	
Avahi_betsil	0	Avahi_betsil	1	Avahi_betsil	this study	1	1225	3.09	987	2.99	0.76	
Avahi_cleesi	1	Avahi_cleesi	1	Avahi_cleese	this study	1	975	2.99	926	2.97	0.95	
Avahi_lanige	1	Avahi_lanige	1	Avahi_lanige	this study	1	1282	3.11	1068	3.03	0.80	
Avahi_merid	0	Avahi_merid	1	Avahi_merid	this study	1	1130	3.05	1043	3.02	0.92	
Avahi_moore	0	Avahi_moore	0	Avahi_moore	this study	1	950	2.98	904	2.96	0.95	
Avahi_occide	1	Avahi_occide	1	Avahi_occide	this study	1	967	2.99	842	2.93	0.85	
Avahi_peyrie	0	Avahi_peyrie	1	Avahi_peyrie	this study	1	1120	3.05	990	3.00	0.87	
Avahi_ramar	0	Avahi_ramar	1	Avahi_ramar	this study	1	1031	3.01	941	2.97	0.90	
Avahi_unicol	1	Avahi_unicol	1	Avahi_unico	this study	1	920	2.96	830	2.92	0.89	
Cheirogaleus	1	Cheirogaleus	1	Cheirogaleus	this study	1	443	2.65	575	2.76	1.30	
Cheirogaleus	1	Cheirogaleus	1	Cheirogaleus	this study	1	143	2.16	132	2.12	0.92	
Daubentonia	1	Daubentonia	1	Daubentonia	this study	1	2446	3.39	2621	3.42	1.07	
Eulemur_alb	1	Eulemur_alb	1	Eulemur_alb	this study	1	2150	3.33	2213	3.34	1.03	
Eulemur_cin	0	Eulemur_cin	1	Eulemur_cin	this study	1	1995	3.30	1923	3.28	0.96	
Eulemur_cor	1	Eulemur_cor	1	Eulemur_cor	this study	1	1080	3.03	1280	3.11	1.19	
Eulemur_flav	1	Eulemur_ma	1	Eulemur_flav	this study	1	2042	3.31	1797	3.25	0.86	
Eulemur_fulv	1	Eulemur_fulv	1	Eulemur_fulv	this study	1	1500	3.18	1600	3.20	1.07	
Eulemur_ma	1	Eulemur_ma	1	Eulemur_ma	this study	1	1987	3.30	1974	3.30	0.99	
Eulemur_mo	1	Eulemur_mo	1	Eulemur_mo	this study	1	1280	3.11	1140	3.06	0.88	
Eulemur_rub	1	Eulemur_rub	1	Eulemur_rub	this study	1	1964	3.29	2067	3.32	1.05	
Eulemur_ruf	0	Eulemur_ruf	1	Eulemur_ruf	this study	1	2232	3.35	2096	3.32	0.94	
Eulemur_ruf	1	Eulemur_ruf	1	Eulemur_ruf	this study	1	1260	3.10	1410	3.15	1.12	
Hapalemur_	1	Hapalemur_	1	Hapalemur_	this study	1	1262	3.10	1235	3.09	0.98	
Hapalemur_	1	Hapalemur_	1	Hapalemur_	this study	1	1500	3.18	1620	3.21	1.08	
Hapalemur_	1	Hapalemur_	1	Hapalemur_	this study	1	838	2.92	838	2.92	1.00	
Hapalemur_	1	Hapalemur_	1	Hapalemur_	this study	1	870	2.94	839	2.92	0.96	
Indri_indri	1	Indri_indri	1	Indri_indri	this study	1	7135	3.85	5825	3.77	0.78	
Lemur_catta	1	Lemur_catta	1	Lemur_catta	this study	1	2268	3.36	2227	3.35	0.98	
Lepilemur_a	1	Lepilemur_a	1	Lepilemur_a	this study	1	920	2.96	832	2.92	0.89	
Lepilemur_a	0	Lepilemur_a	0	Lepilemur_a	this study	1	500	2.70	650	2.81	1.30	
Lepilemur_a	1	Lepilemur_a	1	Lepilemur_a	this study	1	779	2.89	733	2.87	0.94	
Lepilemur_b	0	Lepilemur_b	0	Lepilemur_b	this study	1	1210	3.08	1000	3.00	0.79	
Lepilemur_d	1	Lepilemur_d	1	Lepilemur_d	this study	1	730	2.86	730	2.86	1.00	
Lepilemur_e	1	Lepilemur_e	1	Lepilemur_e	this study	1	983	2.99	982	2.99	1.00	
Lepilemur_g	0	Lepilemur_g	0	Lepilemur_g	this study	1	725	2.86	900	2.95	1.24	
Lepilemur_h	0	Lepilemur_h	0	Lepilemur_h	this study	1	983	2.99	1000	3.00	1.02	
Lepilemur_h	1	Lepilemur_h	0	Lepilemur_h	this study	1	837	2.92	1113	3.05	1.33	

MSW93_Bin	Madagascar	BMassMale	Log10_BMas	BMassFem	Log10_BMas	SSD	SSD_Directio
Allocebus_tr	1	79	1.89762709	76	1.88081359	1.03947368	1
Avahi_lanige	1	1068	3.02857125	1282	3.10788803	0.79962547	0
Brachyuromy	1	113.8	2.05614226	104.86	2.02060985	1.08525653	1
Cheirogaleus	1	575	2.75966784	443	2.64640373	1.2979684	1
Cheirogaleus	1	132	2.12057393	143	2.15533604	0.91666667	0
Cryptoprocta	1	8233.3	3.91557394	7243.8	3.85996645	1.13659957	1
Daubentonia	1	2621	3.41846702	2446	3.38845645	1.07154538	1
Echinops_tel	1	102.4	2.01029996	99.87	1.99943505	1.02533293	1
Eliurus_majo	1	103.88	2.01653194	98.9	1.99519629	1.05035389	1
Eliurus_minc	1	36.22	1.55894845	38.08	1.58069694	0.94864716	0
Eliurus_myo:	1	65.14	1.81384775	65.56	1.81663894	0.99355235	0
Eliurus_tanal	1	87.04	1.93971888	91.14	1.95970902	0.95289522	0
Eliurus_webl	1	78.35	1.894039	80.04	1.90330708	0.97843012	0
Eulemur_cor	1	1280	3.10720997	1080	3.03342376	1.18518519	1
Eulemur_fulv	1	1600	3.20411998	1500	3.17609126	1.06666667	1
Eulemur_ma	1	1974	3.29534715	1987	3.29819787	0.99341439	0
Eulemur_mo	1	1140	3.05690485	1280	3.10720997	0.87719298	0
Eulemur_rub	1	2067	3.31534048	1964	3.29314148	1.05244399	1
Fossa_fossar	1	1530	3.18469143	2289.5	3.35974065	0.50359477	0
Galidia_elega	1	820.4	2.91402565	642.5	2.80787313	1.27688716	1
Galidictis_gra	1	1650	3.21748394	1400	3.14612804	1.17857143	1
Geogale_aur	1	7.34	0.86569606	7.01	0.84571802	1.04707561	1
Gymnuromys	1	136.83	2.13618133	126.07	2.10061175	1.08534941	1
Hapalemur_a	1	1620	3.20951501	1500	3.17609126	1.08	1
Hapalemur_g	1	838	2.92324402	838	2.92324402	1	0
Hapalemur_s	1	2532	3.4034637	2248	3.35179631	1.12633452	1
Hemicentete	1	109.5	2.03941412	107.6	2.03181227	1.01765799	1
Indri_indri	1	5825	3.76529593	7135	3.85339398	0.7751073	0
Lemur_catta	1	2227	3.34772022	2268	3.35564305	0.98158958	0
Macrotarson	1	72	1.8573325	69	1.83884909	1.04347826	1
Microcebus_	1	317	2.50105926	299	2.47567119	1.06020067	1
Microcebus_	1	59	1.77085201	63	1.79934055	0.93220339	0
Microgale_b	1	9.35	0.97081161	9.68	0.98587536	0.96470588	0
Microgale_c	1	12.38	1.09272064	12.69	1.10346162	0.97495961	0
Microgale_d	1	26.93	1.43023635	28.89	1.46074754	0.92721872	0
Microgale_g	1	23.89	1.37821615	22.21	1.34654856	1.0756416	1
Microgale_pa	1	3.08	0.48855072	3.2	0.50514998	0.96103896	0
Microgale_ta	1	38.88	1.58972626	39.69	1.5986811	0.97916667	0
Microgale_th	1	21.72	1.33685982	22.05	1.34340859	0.98480663	0
Mungotictis_	1	560.7	2.74873056	538.7	2.73134698	1.04083906	1
Nesomys_ru	1	163.3	2.21298618	156.64	2.19490267	1.04251788	1
Oryzorictes_	1	37.24	1.57100967	33	1.51851394	1.12848485	1
Phaner_furci	1	328	2.51587384	351	2.54530712	0.92987805	0
Propithecus_	1	6496	3.81264602	6750	3.82930377	0.96089901	0
Propithecus_	1	3390	3.5301997	3590	3.55509445	0.94100295	0
Propithecus_	1	2890	3.46089784	2850	3.45484486	1.01403509	1
Setifer_setos	1	223	2.34830486	218.83	2.34010686	1.01905589	1
Varecia_vari	1	3624	3.55918819	3748	3.57379958	0.96578366	0
Acerodon_ce	0	500	2.69897	280	2.44715803	1.78571429	1
Acinonyx_jul	0	57600	4.76042248	60000	4.77815125	0.95833333	0
Acomys_cah	0	43.3	1.6364879	32	1.50514998	1.353125	1
Acomys_russ	0	44.1	1.64443859	41	1.61278386	1.07560976	1
Aconaemys_	0	133	2.12385164	134	2.1271048	0.9924812	0
Acrobates_p	0	12.3	1.08990511	13	1.11394335	0.94308943	0
Aepyceros_n	0	80000	4.90308999	45150	4.65465775	1.77187154	1
Aethomys_h	0	150.25	2.17681448	135	2.13033377	1.11296296	1
Ailuropoda_	0	139000	5.1430148	97000	4.98677173	1.43298969	1
